# Untargeted Metabolic Profiling of Cat Urine and Plasma in Hypertension

**DOI:** 10.1111/jvim.70227

**Published:** 2025-08-30

**Authors:** Jim Scott‐Baumann, Alice H. Watson, Luis Mur, Harriet M. Syme

**Affiliations:** ^1^ Aberystwyth University Faculty of Earth and Life Sciences, Aberystwyth School of Veterinary Sciences Aberystwyth Wales United Kingdom of Great Britain and Northern Ireland; ^2^ Royal Veterinary College Deptartment Clinical Science and Services Hertfordshire United Kingdom of Great Britain and Northern Ireland

**Keywords:** cardiovascular, feline, hemodynamics, metabolomics, plasma, urineamlodipine

## Abstract

**Background:**

Early diagnosis of hypertension remains an important problem in cats. Lack of routine blood pressure screening in primary care practice, and the possibility of white coat artifact mean the discovery of a new diagnostic test, if less sensitive to short‐term changes in blood pressure associated with veterinary care, would be useful. Identification of metabolomic changes in hypertensive cats could advance understanding of the pathogenesis of hypertension in cats, as well as identify novel biomarkers.

**Objectives:**

Use untargeted metabolomics to identify biochemical changes in cat plasma and urine between normotensive controls (NT) and hypertensive cats before treatment (HTpre); HTpre and hypertensive cats treated with amlodipine (HTtx).

**Animals:**

Biobanked surplus plasma and urine samples were selected from client‐owned cats (> 9 years old) that were NT (urine *n* = 17, plasma *n* = 19), HTpre (urine *n* = 13, plasma *n* = 19), or HTtx (urine *n* = 12, plasma *n* = 19).

**Methods:**

Samples were profiled using flow infusion electrospray—high‐resolution mass spectrometry, and differences assessed using univariate (paired or two sample *t*‐tests) and multivariate (partial least squares discriminant analysis) methods using the R‐based MetaboAnalyst platform. Tentative identifications of metabolites then were made using the MZedDb database.

**Results:**

Significant (false discovery adjusted < 0.01) biochemical differences were observed between each of the sample groups. Biochemical changes in urine between HTpre and NT animals were linked to the tricarboxylic acid cycle, oxidative stress, steroid hormones, taurine metabolism, and phosphatidylinositol‐3,4,5‐trisphosphate.

**Conclusions:**

Metabolites altered in hypertensive cats were similar to those observed in other species.

**Abbreviations**
CKD: chronic kidney disease
HTpre: untreated hypertensive
HTtx: treated hypertensive
m/z: mass:charge ratio
n: negative
NT: normotensive
p: positive
PIP3: phosphatidylinositol‐3,4,5‐trisphosphate
PLSDA: partial least squares discriminant analysis
RAAS: renin angiotensin aldosterone system
TCA cycle: tricarboxylic acid cycle
USG: urine specific gravity

## 1 Introduction

Hypertension is a common but underdiagnosed condition in cats because blood pressure measurement is not routinely performed in primary care practice [1]. Barriers to screening include difficulty in differentiating idiopathic stress‐induced hypertension (i.e., white coat artifact) from pathological hypertension [2], the time required to measure blood pressure, and the cost to the owner [3]. Blood pressure assessment is recommended annually for cats over nine years of age [4], because systolic blood pressure increases with age in cats [5]. Hypertensive cats may not show obvious clinical signs; thus, hypertension is not recognized without routine screening. Left untreated, hypertension can result in target organ damage to the eyes, kidneys, brain, heart, and vasculature [6]. When increased blood pressure is detected, fundic examination may be used to confirm the presence of clinical hypertension by visualizing ocular target organ damage, including retinal bullae, detachment, or hemorrhage [7]. Fundic examination could be used to screen for hypertension in cats but requires time and practice, and lesions are not present in all cases of hypertension [8], especially those detected early.

In humans, hypertension does not usually have an underlying cause and is defined as primary or essential, whereas hypertension in cats often is associated with underlying diseases such as chronic kidney disease (CKD) and hyperthyroidism [9, 10]. Of cats that have CKD, 19%–60% are hypertensive [11, 12]. The underlying pathological mechanisms potentially could be identified by metabolomics, the quantification of thousands of low molecular weight compounds in a biological fluid. Urine metabolomics has been used to identify potential biomarkers for hypertension in humans [13, 14] and rats [15]. Metabolomics is an active area of research in cats, with studies so far having covered diseases such as diabetes mellitus [16], pancreatitis [17], CKD [18, 19, 20], obesity [21], chronic enteropathies [22], gut microbial metabolism [23], and some tumors [24, 25]. Urine metabolomic signatures have been investigated in healthy cats [26], and those with urological disorders [19], but hypertensive cats have yet to be characterized.

Amlodipine is an L‐type, dihydropyridine voltage‐gated calcium channel antagonist that relaxes vascular smooth muscle cells and has been successfully used to treat hypertension in cats for > 25 years [27]. Treatment is usually effective, but if pre‐treatment blood pressure is very high, cats require a higher dose (1.25 mg daily) to decrease blood pressure to < 160 mmHg [28]. Interestingly, when amlodipine is given to healthy young cats, even at 5 times the recommended dose, no change in blood pressure is observed, illustrating that the drug works by reversing arterial constriction and, if not present, no effect occurs [29]. Although amlodipine preferentially relaxes the afferent arteriole, risking the development or exacerbation of glomerular hypertension, it has been shown to significantly decrease proteinuria when given to cats with naturally occurring systemic hypertension, probably because the decrease in systemic blood pressure is so marked [30]. The effect of amlodipine on serum and urine metabolomes has not been investigated in cats or in humans.

We hypothesized that plasma and urine metabolites would differ between normotensive and hypertensive cats and between untreated hypertensive and amlodipine‐treated hypertensive cats. Differences between normotensive and hypertensive cats could provide useful insight into disease pathogenesis or identify potential new biomarkers with diagnostic potential. Such biomarkers could be used for initial diagnosis and could also be used for ongoing monitoring of response to treatment. Simultaneous evaluation of blood and urine samples may be advantageous because metabolites present in the urine will reflect processes ongoing for several hours before sample collection, whereas changes in blood may be more affected by transient alterations, such as those associated with the stress of handling the cat. When concordant alterations are evident in the metabolome from multiple different matrices, this finding can increase confidence in the likely clinical relevance of the observed changes. In addition, the metabolome of urine is typically less complex than that of blood, which may be an advantage. Changes in the urine profile also may be indicative of alterations in renal function that cannot be detected in plasma and may be pertinent because CKD is a major risk factor for the development of hypertension in cats [5].

## 2 Materials and Methods

### 2.1 Case Selection

Cases were retrospectively selected from an ongoing longitudinal study of 4021 client‐owned neutered cats > 9 years of age from two first opinion practices in central London (People's Dispensary for Sick Animals, Bow and Beaumont Sainsbury Animal Hospital, Camden) between 2011 and 2021.

Clinical records were reviewed to select hypertensive cats diagnosed with systolic blood pressure > 160 mmHg and with concurrent hypertensive retinal lesions on indirect fundic examination. Systolic blood pressure was measured following a standardized clinic protocol after brief acclimatization, using a Doppler technique with an average of five readings taken during each examination. No sedatives or anxiolytic medications were given, and cats receiving medications other than routine parasiticides were excluded from the study. Hypertensive cases had blood samples collected at the time of diagnosis and after treatment with amlodipine (0.625–2.5 mg daily). Follow‐up samples were collected within 42 days of starting treatment when blood pressure was < 160 mmHg. Normotensive (NT) controls with blood pressures < 140 mmHg were selected by stratified random sampling based on matching serum creatinine concentration, age, sex, and breed between hypertensive cases and controls (Table 1). Fundic examination was not performed in normotensive cats. All cats with hyperthyroidism, urinary tract infection, or those receiving anti‐hypertensive medications other than amlodipine were excluded. Diet was not specifically controlled between groups, but all cats with azotemic CKD were offered a renal diet, independent of hypertension status.

**TABLE 1:** Metadata from normotensive (NT, *n* = 19), hypertensive pre‐treatment (HTpre, *n* = 19) and hypertensive treated (HTtx, *n* = 19) cats that had plasma samples compared using untargeted metabolomics.

Variable		NT (*n* = 19)	HTpre (*n* = 19)	HTtx (*n* = 19)	*p*
Sex	Male	10	10		1.00
	Female	9	9		
Breed	DSH	15	14		1.00
	DLH	2	2		
	Burmese/Burmese X	1	2		
	BSH	1	1		
Age	(years)	15.0 [9.5–20.0]	14.6 [10.0–20.1]	14.6 [10.0–20.1]	0.86
Creatinine	(mg/dL) (μmol/L)	2.01 [1.15–3.20] 178 [102–283]	2.04 [1.05–3.51] 180 [93–310]	1.96 [0.85–2.92] 173 [75–258]	0.84
Weight	(kg)	3.65 [2.46–5.31]	3.68 [2.82–5.95]	3.87 [2.83–5.85]	0.45
BP	(mmHg)	122 [96–139]	191 [172–234]	142 [118–154]	**< 0.001**
USG		1.023 [1.016–1.041]	1.018 [1.011–1.036]	1.017 [1.008–1.044]	**0.02**
Potassium	(mEq/L)	4.1 [2.8–5.2]	3.7 [3.2–4.2]	3.8 [3.2–4.4]	0.16

*Note:* Similar results when subsetted to include only cats which had urine analyzed. Continuous data are presented as median [range]. *p*‐value represents comparison between NT and HTpre groups using Fisher's exact test for categorical variables and Kruskal–Wallis test for continuous variables. X indicates cross breed. Bolding indicates significant *P* < 0.05.

Abbreviations: BP, blood pressure; USG, urine specific gravity.

### 2.2 Sample Collection

Blood samples were collected by jugular venipuncture, and urine samples were collected by cystocentesis when the bladder was palpable. Samples were stored in a cooled bag for up to 6 h before processing. Urine specific gravity measurement was performed using a refractometer, alongside dipstick analysis and sediment examination. Blood and urine were centrifuged at 1962 × *g* for 10 min at 4°C. Heparinized plasma was submitted to an external laboratory (IDEXX laboratories, Wetherby, UK) for biochemical analysis, and residual supernatant plasma and urine were stored at −80°C until analysis. Samples were collected with the informed consent of owners and with approval from the Ethics and Welfare Committee of the Royal Veterinary College (URN 2013 1258 and URN 2013 1258E).

### 2.3 Flow Infusion Electrospray High Resolution Mass Spectrometry (FIE‐HRMS)

All samples were processed for metabolomics in a blinded manner. Two hundred μL of heparinized plasma or urine was added to 1520 μL of 4:1 (v/v) mixture of methanol: chloroform (high‐performance liquid chromatography grade) and 50 mg of acetone‐washed glass beads (< 160 μm, Sigma, UK). Samples were vortexed, shaken (15 min at 4°C), left to settle (−80°C for 20 min) with glass beads (< 100 μm diameter) to disrupt cellular material, and then centrifuged at 1800 × *g* for 10 min at 3°C. One hundred μL of the supernatant was transferred to a glass vial with glass insert for analysis in the mass spectrometer. Flow injection electrospray high‐resolution mass spectrometry does not involve prior chromatographic separation, as used in gas chromatography‐mass spectrometry. Sample injection order was randomized to minimize batch effects; master mixes of the entire sample batch also were included throughout the sample run to allow calibration and to correct for any instrument drift. Samples were profiled on an ExactiveTM Orbitrap Mass Spectrometer (Thermo Scientific) as described previously [31]. Samples (20 μL volume) were injected into a flow of 100 μL/min methanol: water (70:30, v/v). Ion intensities were acquired between a scan range of mass to charge ratios (m/z) 50 to 1000 for 3.5 min at a resolution setting of 100,000 (at m/z 200) resulting in 3 (±1) parts per million mass accuracy. A spectral binning approach was applied using BinneR, which eliminated anomalous single scan m/z events and generated averaged spectra across the infusion profile. The modal accurate m/z then was extracted for each bin [32]. The m/z data were normalized based on total ion count using the R package metabolyseR v0.14.10 [33]. The m/z are reported with a prefix to indicate negative (n) or positive (p) charge.

### 2.4 Statistical Analysis

The R software (R version 4.2.2) was used for statistical analysis of clinicopathologic data. Fisher's exact tests were used for categorical data (breed and sex). Histograms and Shapiro Wilk tests were used to examine the distribution of continuous data. For continuous data, non‐parametric Kruskal–Wallis tests with post hoc pairwise Wilcoxon signed rank sum tests with Benjamini‐Hochberg correction were applied, and significance was set at *p* < 0.05.

Graphical and statistical analysis of metabolomics data were performed using the MetaboAnalyst 4.0 platform [34]. Urine metabolite quantification was normalized using urine specific gravity (USG) to correct for urine osmolality; each m/z intensity value was normalized to a USG of 1.015. Raw data were 40% filtered based on interquartile range because there were > 1000 variables [35], log_10_ transformed, and Pareto‐scaled before analysis. Data normality were assessed by visualization in a histogram after transformation and before analysis. Partial least squares discriminant analysis (PLSDA) was validated using training and test sets, where the results were accepted when *Q* ^2^ was > 0.6 and leave‐one‐out cross‐validation accuracy was > 0.6. Individual m/z present at significantly different amounts between groups (adjusted *p* < 0.01) were identified using *t‐*tests (either paired or two sample) and Bonferroni‐corrected for multiple comparisons. The adjusted *p*‐value of 0.01 refers to the false discovery rate based on Bonferroni correction, which is based on 10 independent tests using the original significance level (*p* = 0.05). Paired plasma (*n* = 19) and urine (*n* = 9) samples from cats with untreated hypertension (HTpre) and cats on treatment for hypertension (HTtx) were analyzed using paired *t*‐tests. NT and HTpre groups were compared using two‐sample *t*‐tests. Significant m/z then were tentatively identified using the MZedDb database (http://mzeddb.ibers.aber.ac.uk/) based on predicted masses m/z identified and likely ionization forms [36], m/z to metabolite conversions were based on accurate masses (mass tolerance = 5 part per million resolution). The following adducts: [M^+^]^+^, [M + H]^+^, [M + NH_4_]^+^, [M + Na]^+^, [M + K]^+^, [M‐NH_2_ + H]^+^, [M‐CO_2_H + H]^+^, [M‐H_2_O + H]^+^; [M−]^−^, [M − H]^−^, [M + Na −2H]^−^, [M + Cl]^−^, [M + K −2H]^−^ as well as isotopes were considered. Correlations between multiple adducts of the suspected metabolites were used in the identification process. This tentative identification cannot distinguish between isomers or metabolites with identical molecular masses. Where a m/z could not be identified as a specific metabolite, it is reported as the m/z ratio. Heat maps compared the relative amounts of the 25 most significant metabolites based on *t*‐test. Log_2_‐fold changes were calculated for each significant metabolite and can be found in Table S1.

## 3 Results

### 3.1 Case Selection

Plasma was available from 19 NT cats, and 19 HTpre and HTtx cats. Groups were matched for breed, sex, and serum creatinine concentration. Urine samples were available from 17 NT, 13 HTpre, and 12 HTtx cats; nine hypertensive cats had paired HTpre and HTtx samples. Of the HTtx cats, 11/19 required 0.625 mg amlodipine once daily, whereas one cat required 1.25 mg twice daily to control blood pressure. No differences in age (*p* = 0.86), urine specific gravity (*p* = 0.02), serum potassium concentration (*p* = 0.16) or diet fed (renal or maintenance, *p* = 0.11) were observed between groups (Table 1). As expected, blood pressure was different among each of the three groups (*p* < 0.001) with median values of 122 mmHg for the NT group, 191 mmHg for the HTpre group, and 142 mmHg for the HTtx group.

### 3.2 Flow Infusion Electrospray High Resolution Mass Spectrometry

**FIGURE 1:**
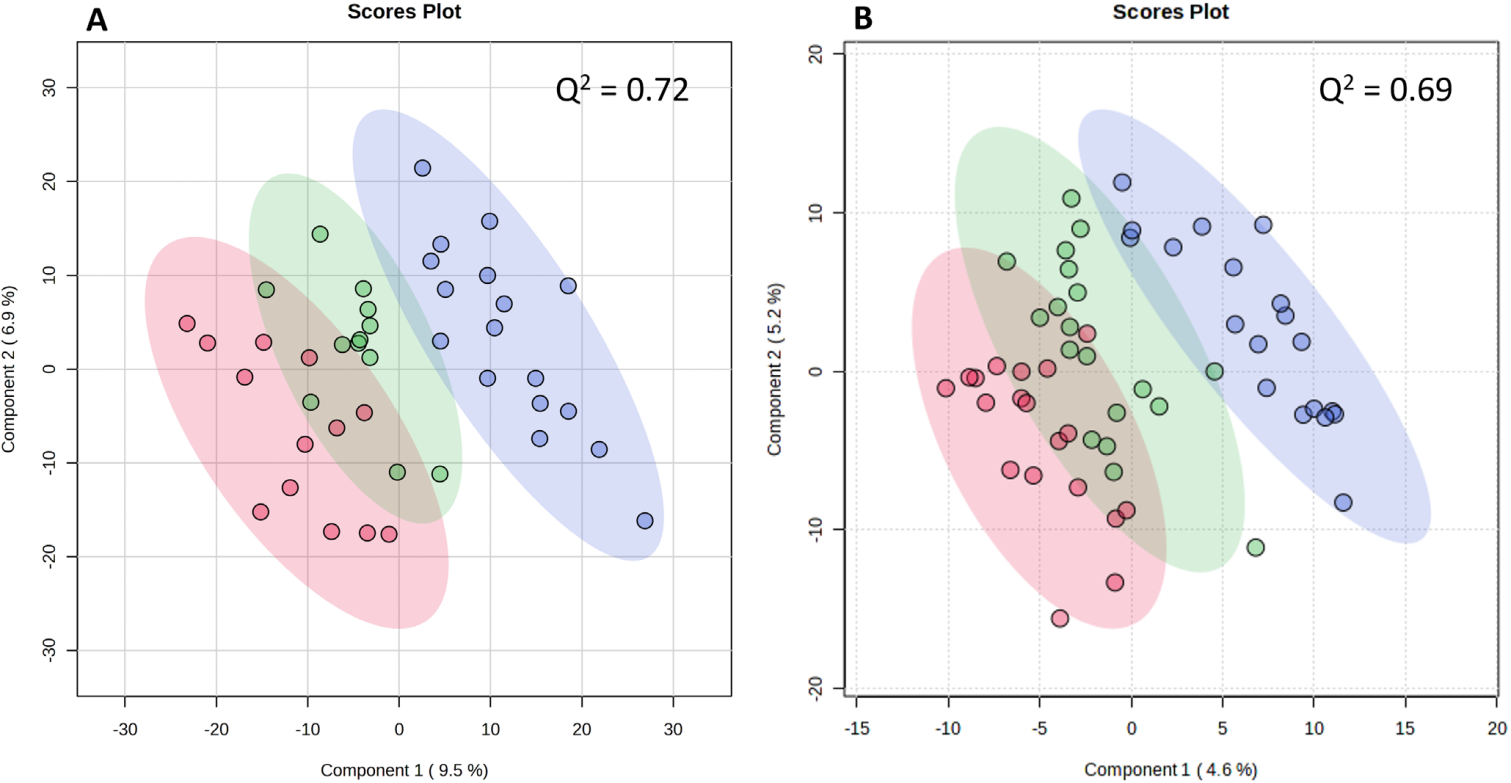
Partial least squares discriminant analysis of feline urine samples (A, *n* = 42) and plasma samples (B, *n* = 67) comparing untreated hypertensive (HTpre, red), amlodipine treated hypertensive (HTtx, green) and normotensive cats (NT, blue). The shaded circles give 95% confidence intervals for each group.

Clustering of points in the PLSDA showed modest separation of the three experimental groups in plasma (Figure 1A) and urine samples (Figure 1B). In plasma, one metabolite (n75.01537—unidentified) was significantly lower in the HTpre than HTtx groups (*p* = 4.17 × 10^−7^, log_2_(FC) = −1.91) and no metabolites were different between the HTpre and NT groups. In urine, a different metabolite (p774.82532—unidentified) was significantly higher in the HTpre than HTtx groups (*p* = 5.30 × 10^−6^, log_2_(FC) = 0.39); 498 metabolites were present at significantly different amounts between the HTpre and NT groups (Table S1).

**FIGURE 2:**
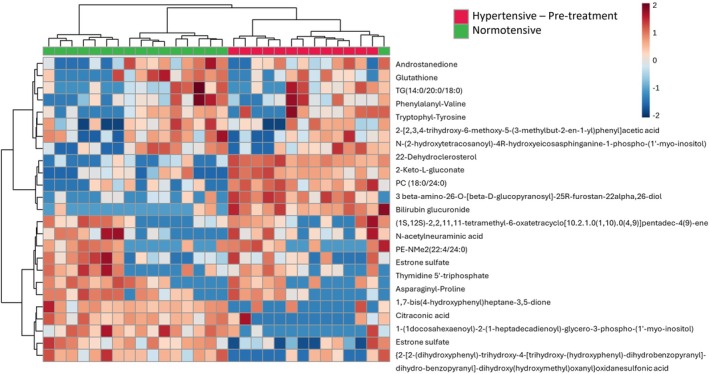
Heatmap comparing the relative amounts of the of the top 25 most significantly altered metabolites (*P* < 0.01) in urine samples of cats with untreated hypertension (HTpre, red, *n* = 13) and normotensive cats (NT, green *n* = 17). For each metabolite (rows) red depicts higher and blue depicts lower amounts. Each column represents a sample from a different cat.

Metabolites present at different amounts in urine between the HTpre and NT groups included higher amounts of phosphatidylinositol‐3,4,5‐trisphosphate (*p* = 2.85 × 10^−4^, log_2_(FC) = 1.78) and lower amounts of amino acid derivatives including prolyl‐arginine [*p* = 1.89 × 10^−4^, log_2_(FC) = −1.64], N‐ornithyl‐L‐taurine [*p* = 1.86 × 10^−4^, log_2_(FC) = −0.59] and glutathione [*p* = 2.07 × 10^−5^, log_2_(FC) = −2.01], uremic toxins (p‐cresol sulfate) [*p* = 9.91 × 10^−4^, log_2_(FC) = −1.40] and indoxyl‐sulfate [*p* = 1.82 × 10^−3^, log_2_(FC) = −1.31], and components associated with the tricarboxylic acid (TCA) cycle including citraconic acid [*p* = 3.04 × 10^−5^, log_2_(FC) = −1.01], oxalosuccinate [*p* = 4.68 × 10^−4^, log_2_(FC) = −1.03], N‐methylphenylalanine [*p* = 7.87 × 10^−4^, log_2_(FC) = −1.29], and phenylalanyl‐valine [*p* = 6.20 × 10^−5^, log_2_(FC) = −5.39], (Figure 2). Tentatively identified steroids including estrone glucuronide (*p* = 1.40 × 10^−4^, log_2_(FC) = −1.44), 19‐noraldosterone (*p* = 4.63 × 10^−5^, log_2_(FC) = −2.72) and androstanedione (*p* = 6.16 × 10^−5^, log_2_(FC) = −2.28) were lower in the HTpre group than in the NT group; whereas estrone sulfate (*p* = 1.23 × 10^−6^, log_2_(FC) = 2.46), 11‐oxo‐androsterone glucuronide (*p* = 3.04 10^−4^, log_2_(FC) = 1.19), testosterone glucuronide (*p* = 3.69 10^−4^, log_2_(FC) = 1.31), and 4‐hydroxyandrostenedione glucuronide (*p* = 5.37 × 10^−4^, log_2_(FC) = 1.41) all were higher in the HTpre group than in the NT group. None of the significantly altered metabolites found in plasma were also found to be significantly altered in urine.

## 4 Discussion

Partial least squares discriminant analysis showed separation among the three groups and provides proof‐of‐concept that untargeted metabolomics can differentiate between cats based on their hypertension status. More individual significant m/z (and their tentative metabolite identifications) were detected in urine samples than in plasma, and resulted in more separation of the groups in the PLSDA plot. These differences could be associated with renally‐produced metabolites in urine, given the central role the kidneys play in the development of hypertension. Although urine has lower relative complexity than blood, > 4500 metabolites have been reported in urine, and discrete biochemical changes have been linked to obesity, cancer, inflammation, and neurological disease in humans [35]. Multiple methods have been described for the normalization of urine samples to correct for the effect of variable volume. No universal agreement exists regarding the best method of correction because results have varied among studies [36, 37]. In our study, urine was normalized to specific gravity, which is routinely used for clinical samples.

Relatively few significant differences were observed in the metabolome of hypertensive cats before and after treatment with amlodipine. This result was obtained despite the fact that paired comparison often is more powerful in detecting differences in clinical samples because each individual (its genetics and environment) serves as its own control. This finding may suggest that other than causing arteriolar dilatation and thus decreasing blood pressure, amlodipine had relatively little effect on treated cats. This finding could be seen as either an advantage (few adverse effects resulting in a good safety profile) or a disadvantage (failure to modulate the underlying pathophysiology of hypertension in CKD). These findings support clinical data that show that treatment with amlodipine effectively decreases blood pressure and decreases the risk of target organ damage but does not affect the progression of CKD or alter patient survival times [28, 38]. The PLSDA showed that amlodipine treatment of the hypertensive cats resulted in only moderate separation from the pre‐treatment group with overlap remaining, and they remained separate from the normotensive group, suggesting that underlying differences between normotensive and hypertensive cats were more substantial and not markedly affected by drug treatment. These results parallel those observed in humans, where treatment with anti‐hypertensive agents, including amlodipine, results in relatively little change to metabolic profiles [39].

Urinary metabolites derived from amino acids including prolyl‐arginine (*p* = 1.89 × 10^−4^, log_2_(FC) = −1.64), N‐ornithyl‐L‐taurine (*p* = 1.86 × 10^−4^, log_2_(FC) = −0.594) and glutathione (*p* = 2.07 × 10^−5^, log_2_(FC) = −2.015) were present at lower amounts in HTpre than NT cats. These, or related metabolites, also are altered in hypertension in other species [15, 40, 41, 42]. Prolyl‐arginine is a dipeptide formed from L‐proline and L‐arginine. Arginine is involved in the nitric oxide (NO) pathway and the renin angiotensin aldosterone system (RAAS) [43], and arginine is effective in decreasing blood pressure in humans [40]. Arginine is an essential amino acid in cats [44], and thus a variation in dietary arginine could explain the observed differences because cats in our study were fed a range of commercial diets, and diet was not controlled. N‐ornithyl‐L‐taurine consists of taurine with a linked ornithine, and taurine is another essential amino acid in cats [45] that decreases blood pressure in spontaneously hypertensive rats [15] and in humans with essential hypertension [41]. Urinary taurine is associated with diet in cats [19], but is not dependent on CKD status [20], and a link with hypertension status has not been identified previously. Glutathione is associated with taurine and hypotaurine metabolism and is a vascular antioxidant [46]. Depletion of glutathione causes hypertension in rats [41], and glutathione is decreased in humans with hypertension [42]. Glutathione, prolyl‐arginine, and N‐ornithyl‐L‐taurine amounts did not differ among plasma samples in our study. This observation could suggest that differences in the urine metabolome arise because of local effects in the kidney, such as excretion. Alternatively, this finding could have been a result of cats being fed different diets because we studied a heterogeneous population of client‐owned cats fed commercial diets, and diets were not matched between groups. Commercial diets are not all equivalent, and diet has been shown to affect the urine metabolome [19, 47]. The NT group included more cats being fed a renal diet (*n* = 6) compared with the HTpre group (*n* = 1). Wet and dry diets can influence metabolomes [19], but information on whether the diets were wet or dry was not available. In addition, although owners were requested to fast their cats for 8 h before clinical examination and sampling, compliance with this recommendation may have been inconsistent, potentially introducing an additional source of variability into the study.

Amounts of the uremic toxins p‐cresol sulfate (*p* = 9.91 × 10^−4^, log_2_(FC) = −1.403) and indoxyl sulfate (*p* = 1.8 × 10^−3^, log_2_(FC) = −1.31) were lower in the urine of the HTpre than in the NT group, but the link of these toxins with hypertension is unclear. P‐cresol sulfate is a gut‐derived uremic toxin, which increases in humans as renal function worsens [48], and may promote renal fibrosis by increasing production of reactive oxygen species and activation of RAAS [49, 50]. Lower amounts of indoxyl sulfate in the HTpre group were unexpected because indoxyl sulfate is associated with stimulation of the intrarenal RAAS in mice [50]. Furthermore, both metabolites induce oxidative stress, which is increased in hypertension and affects the viability of vascular endothelial cells [51, 52]. Alterations in uremic toxins may occur because of shifts in the gut microbiome, but lower amounts would be associated with decreased oxidative stress, which is the opposite of the situation in hypertension.

One confounding factor that could affect the amount of uremic toxins is renal function because amounts in cat blood and feces are reportedly increased in CKD [53, 54]. Chronic kidney disease is a common co‐morbidity in hypertensive cats and results in changes in the serum metabolome [20], although one small study showed no significant impact of CKD on the urine metabolome [19]. Other studies have shown significant shifts in the urine metabolome of cats with CKD [55], even before azotemia develops [56]. To account for the potential confounding effect of CKD in our study, the NT and HT groups were matched for the severity of azotemia. Neither p‐cresol sulfate nor indoxyl sulfate was different in plasma between experimental groups in our study. Other factors likely to affect the amounts of these uremic toxins include antibiotic use [57], diet, and intestinal transit time [58], because all of these have effects on gut microbiota. Furthermore, cats fed betaine and prebiotics have decreased serum concentrations of p‐cresol sulfate [59]. One study has suggested associations between hypertension and gut microbiota in species other than cats. These cover many proposed mechanisms (sympathetic nervous system activation, chronic inflammation, endothelial dysfunction) and many are yet to be verified [60]. Investigation of the microbiome of cats during hypertension therefore also would be of interest.

Some metabolites linked to the TCA cycle (including citraconic acid [*p* = 3.04 × 10^−5^, log_2_(FC) = −1.01], oxalosuccinate [*p* = 4.68 × 10^−4^, log_2_(FC) = −1.03], N‐methylphenylalanine [*p* = 7.87 10^−4^, log_2_(FC) = −1.29], and phenylalanyl‐valine [*p* = 6.20 × 10^−5^, log_2_(FC) = −5.39]) also were lower in urine from the HTpre group than the NT group. Both oxalosuccinate and citraconate have anti‐oxidative properties [61], and succinate, derived from oxalosuccinate, is a hypertensive agent that acts through modulation of the RAAS [62]. Therefore, amounts of these metabolites may be expected to be higher in hypertension rather than lower, as found in our study. Phenylalanine is a component of both methylphenylalanine and phenylalanyl‐valine. Phenylalanine enters the TCA cycle, and microbial fermentation of phenylalanine produces P‐cresol sulfate [63], which also was significantly decreased in the urine of the HTpre group.

Phosphatidylinositol‐3,4,5‐trisphosphate (PIP3) is a phospholipid responsible for many downstream signaling components involved in cell growth and survival. Data from mouse models suggest that PIP3 mediates aldosterone‐induced epithelial sodium channel activity, which could lead to excessive sodium retention and resulting hypertension [64]. Amounts of PIP3 were higher in HTpre than in NT cats (*p* = 2.85 × 10^−4^, log_2_(FC) = 1.78). Exogenous PIP3, when applied to vascular myocytes, replicates the Ca^2+^ channel‐stimulating effect of angiotensin II and phosphoinositide 3‐kinase, illustrating its importance in maintaining vascular tone [65].

The amounts of multiple steroid hormones or their downstream products were significantly different in the urine of the HT‐pre and NT groups. These included 19‐noraldosterone, which was lower in the urine of the HT‐pre group when compared with the NT group (*p* = 4.63 × 10^−5^, log_2_(FC) = −2.72). In humans, this mineralocorticoid hormone is increased in the urine of some hypertensive patients, but these changes are not consistent [66]. Identification of steroid hormones from metabolomic data is difficult because > 1 steroid hormone may have the same m/z. Although mass spectrometry can be used for the identification and quantification of steroid hormones, it involves the use of validated protocols and the inclusion of individual hormone standards to allow targeted profiling and quantification of specific metabolites. In contrast, we used an untargeted, semi‐quantitative approach.

Our study demonstrated that untargeted metabolomics can differentiate between cats with and without hypertension. However, the untargeted nature of the methodology makes it difficult to identify each metabolite contributing to these differences. Future work should focus on using authentic standards for targeted profiling to more confidently identify and quantify metabolites of interest. Studies of this kind have high dimensionality (the number of features is much higher than the sample size), and a power calculation was not performed before the study given the small number of samples available. However, our study is likely under‐powered, and additional studies of this kind should use larger sample sizes. A larger study also would allow further analysis through stratification of the patients by their clinical variables (e.g., by serum creatinine or potassium concentrations), which was not performed in our study. Fecal microbiome analyses could help identify any significant shifts in gut bacteria, such as bacteria producing uremic toxins that could be responsible for the changes in P‐cresol sulfate and indoxyl sulfate seen in our study, as has been done for cats with CKD [54]. Sampling of tissue at necropsy from cats with hypertension could help identify changes in the kidneys using metabolomics or transcriptomics and could utilize tissue samples taken shortly after euthanasia. A prospective repeated measures study, where metabolomic analysis of the same sample types from healthy cats recruited into the study, where some later go on to develop hypertension, also would be useful to confirm our findings.

## Disclosure

Authors declare no off‐label use of antimicrobials.

## Ethics Statement

Approval by Royal Veterinary College to cover the collection and storage of (residual) blood and urine after samples are collected for diagnostic purposes in a longitudinal health screening of mature, senior, and geriatric cats (URN: 2013 1258E). Authors declare human ethics approval was not needed.

## Conflicts of Interest

The authors declare no conflicts of interest.

## Supporting information


**Table S1:** Metabolites significantly altered in the urine of cats when comparing untreated hypertensives (HT‐pre, *n* = 13) with normotensives (NT, *n* = 17).
