# Supplementary material for: Untargeted Metabolic Profiling of Cat Urine and Plasma in Hypertension
Source: J Vet Intern Med. 2025 Aug 30;39(5):e70227. doi: 10.1111/jvim.70227 (PMC12397984; doi:10.1111/jvim.70227)
Supplement: Supplementary file 1 — Table S1: Metabolites significantly altered in the urine of cats when comparing untreated hypertensives (HT‐pre, n = 13) with normotensives (NT, n = 17). [file JVIM-39-e70227-s001.docx]

**Supplementary material**

**Table S1:** Metabolites significantly altered in the urine of cats when comparing untreated hypertensives (HT-pre, n=13) with normotensives (NT, n=17).

| **ID** | **m/z** | **log2(fold change)** | **p value** | **higher or lower in hypertensives compared to normotensives** |
| --- | --- | --- | --- | --- |
| N-(2-hydroxytetracosanoyl)-4R-hydroxyeicosasphinganine-1-phospho-(1'-myo-inositol) | n976.66315 | 2.8511 | 1.11E-07 | higher |
| PC(o-18:0/24:0) | n897.70648 | 3.1748 | 1.36E-07 | higher |
| 1-(2,4-dihydroxyphenyl)-3-(5-methoxy-2,2-dimethyl-2H-chromen-6-yl)propan-1-one | n393.08966 | -3.9796 | 1.48E-07 | lower |
| unidentified | p724.83502 | 2.2073 | 1.74E-07 | higher |
| unidentified | n712.784 | 2.9423 | 2.11E-07 | higher |
| (2Z,4Z)-2,3,4,5-tetrachlorohexa-2,4-dienedioic acid | n316.81741 | -2.3096 | 3.51E-07 | lower |
| TG(14:1(9Z)/22:6(4Z,7Z,10Z,13Z,16Z,19Z)/o-18:0) | n900.70947 | 3.3689 | 4.34E-07 | higher |
| 1,2-diheptadecanoyl-3-11Z-docosenoyl-sn-glycerol | p468.94278 | 1.4042 | 7.95E-07 | higher |
| 9,10,12,13-tetrabromo-octadecanoic acid | p636.88495 | 3.2496 | 9.29E-07 | higher |
| 1-nonadecanoyl-glycero-3-phospho-(1'-sn-glycerol) | p1074.63452 | 3.3907 | 1.04E-06 | higher |
| Estrone sulfate | p198.04901 | 2.4622 | 1.23E-06 | higher |
| bacteriohopane-,32,33,34-triol-35-(N-(9-cyclohexyl-nonanoyl))-glucosamine | n968.67712 | 3.0041 | 1.34E-06 | higher |
| (2E,6Z)-3,7,11-trimethyldodeca-2,6,10-trien-1-yl trihydrogen diphosphate | n382.12811 | -2.3652 | 1.67E-06 | lower |
| (S)-(E)-8-(3,6-Dimethyl-2-heptenyl)-4',5,7-trihydroxyflavanone | p433.16043 | -1.4862 | 1.89E-06 | lower |
| Fucalpha1-2Galbeta1-3GlcNAcbeta1-3(GlcNAcbeta1-6)Galbeta1-3GlcNAcbeta1-3Galbeta1-4Glcbeta-Cer(d18:1/24:0) | p1036.59497 | 2.5699 | 2.46E-06 | higher |
| unidentified | n769.7594 | 2.8613 | 3.53E-06 | higher |
| 3,4,5-trihydroxy-6-{[1-(4-methoxyphenyl)-4-methylpentan-3-yl]oxy}oxane-2-carboxylic acid | n424.1376 | -1.9459 | 5.72E-06 | lower |
| unidentified | n483.85532 | 1.7337 | 5.93E-06 | higher |
| 10R,17S-dihydroxy-4Z,7Z,11E,13E,15E,19Z-docosahexaenoic acid | p181.12271 | 2.2249 | 5.95E-06 | higher |
| DG(24:0/0:0/24:1n9) | n789.73694 | 2.3552 | 6.45E-06 | higher |
| 2-Keto-L-gluconate | n411.05615 | 2.4906 | 8.9E-06 | higher |
| Asparaginyl-Proline | p230.11374 | 2.2359 | 8.99E-06 | higher |
| 1-(4Z,7Z,10Z,13Z,16Z,19Z-docosahexaenoyl)-2-(9Z,12Z-heptadecadienoyl)-glycero-3-phospho-(1'-myo-inositol) | n296.8345 | -1.9522 | 9.17E-06 | lower |
| 3beta-amino-26-O-[beta-D-glucopyranosyl]-25R-furostan-22alpha,26-diol | n617.38666 | 1.4405 | 9.97E-06 | higher |
| N-Acetylneuraminic acid | p348.05215 | 1.499 | 1.02E-05 | higher |
| (1S,12S)-2,2,11,11-tetramethyl-6-oxatetracyclo[10.2.1.0(1,10).0(4,9)]pentadec-4(9)-ene | p299.17722 | 4.0156 | 1.07E-05 | higher |
| N-(hexacosanoyl)-1-beta-glucosyl-sphinganine (aka GlcCer(d18:0/26:0)) | n840.72974 | 2.6724 | 1.07E-05 | higher |
| GalNAcbeta1-3Galalpha1-3Galalpha1-3Galalpha1-3Galalpha1-4Galbeta1-4Glcbeta-Cer(d18:1/26:0) | n617.3454 | -2.2563 | 1.11E-05 | lower |
| bacteriohopane-,32,33,34-triol-35-(N-(9-cyclohexyl-nonanoyl))-glucosamine | p947.77557 | 2.0467 | 1.16E-05 | higher |
| 1,2-di-(9Z,12Z-heptadecadienoyl)-3-octadecanoyl-sn-glycerol | p877.72919 | 2.0962 | 1.28E-05 | higher |
| NA | p478.16739 | -1.9272 | 1.45E-05 | lower |
| 2-O-hexadecanoyl-3-O-(2S,4S,6S-trimethyl-3S-hydroxy-tricosanoyl)-alpha,alpha-trehalose | p1052.6532 | 2.2413 | 1.66E-05 | higher |
| (25R)-5beta-cholestane-3alpha,7alpha,12alpha,26-tetrol | n871.69904 | 2.1294 | 1.71E-05 | higher |
| unidentified | n436.88818 | -1.7675 | 1.74E-05 | lower |
| O-b-D-Gal-(1->3)-O-[O-b-D-Gal-(1->4)-2-(acetylamino)-2-deoxy-b-D-Glc-(1->6)]-2-(acetylamino)-2-deoxy- D-Galactose | p376.14377 | 2.3719 | 1.89E-05 | higher |
| PE-NMe2(22:4(7Z,10Z,13Z,16Z)/24:0) | p954.67444 | 3.0137 | 1.94E-05 | higher |
| Bilirubin glucuronide | p381.15469 | 2.1211 | 2E-05 | higher |
| 2-[2,3,4-trihydroxy-6-methoxy-5-(3-methylbut-2-en-1-yl)phenyl]acetic acid | p300.14291 | -2.0331 | 2E-05 | lower |
| 1,7-bis(4-hydroxyphenyl)heptane-3,5-dione | n311.1286 | -1.2247 | 2.04E-05 | lower |
| Glutathione | p346.04651 | -2.0155 | 2.08E-05 | lower |
| unidentified | p739.86615 | -1.706 | 2.28E-05 | lower |
| 1-[2,4-dihydroxy-6-methoxy-3-(3-methylbut-2-en-1-yl)phenyl]-3-phenylpropan-1-one | n377.13361 | -2.3669 | 2.7E-05 | lower |
| 3beta-Acetoxy-4-oplopanone | p297.20642 | 1.5298 | 2.96E-05 | higher |
| 16-Hydroxy-3-oxo-12-oleanen-28-oic acid | n939.66956 | 1.8927 | 3.04E-05 | higher |
| Citraconic acid | n151.0013 | -1.0102 | 3.04E-05 | lower |
| 2-(2,6-dihydroxyphenyl)-4-methyl-6-(2,4,5-trihydroxyphenyl)cyclohex-3-ene-1-carboxylic acid | p395.11014 | -1.2838 | 3.09E-05 | lower |
| 7-[7,8,9,12,13,14,17,18,19,25-decahydroxy-24-(hydroxymethyl)-4,22,27-trioxo-3,23,26-trioxahexacyclo[13.10.3.1Â²,â¶.0âµ,Â¹â°.0Â¹Â¹,Â²â¸.0Â¹â¶,Â²Â¹]nonacosa-5(10),6,8,11,13,15(28),16,18,20-nonaen-29-yl]-3,4,8,9,10-pentahydroxy-6-oxo-6H-benzo[c]chromene | p490.02487 | 1.8538 | 3.11E-05 | higher |
| GalNAcalpha1-3(Fucalpha1-2)Galbeta1-3GalNAcalpha1-3(Fucalpha1-2)Galbeta1-4GlcNAcbeta1-3Galbeta1-4Glcbeta-Cer(d18:1/22:0) | p1108.5802 | 2.1346 | 3.13E-05 | higher |
| (5Z,7E)-(1S,3R)-23,24-dinor-9,10-seco-5,7,10(19)-cholatriene-1,3,22-triol | p347.25882 | 1.5502 | 3.6E-05 | higher |
| {2-[4-(4-chloro-1,2-diphenylbut-1-en-1-yl)phenoxy]ethyl}(methyl)amine | n429.13977 | -1.8663 | 3.6E-05 | lower |
| 1-hexadecyl-2-tetradecanoyl-glycero-3-phospho-(1'-sn-glycerol) | p719.46368 | 1.9037 | 3.8E-05 | higher |
| 2-{[hydroxy(6-hydroxy-7-methoxy-2H-1,3-benzodioxol-5-yl)methylidene]amino}acetic acid | p306.01767 | -1.6741 | 3.96E-05 | lower |
| Cysteinylglycine | p162.02208 | 1.508 | 4.06E-05 | higher |
| 1,2-dihexadecanoyl-3-(9Z-heptadecenoyl)-sn-glycerol | n856.70502 | 2.2018 | 4.36E-05 | higher |
| 1-(9Z-heptadecenoyl)-2-tridecanoyl-glycero-3-phosphate | n309.20871 | 1.4005 | 4.39E-05 | higher |
| unidentified | n1028.65698 | 1.85 | 4.41E-05 | higher |
| 3,4,5-trihydroxy-6-(3-{3,5,7-trihydroxy-6-[3,4,5-trihydroxy-6-(hydroxymethyl)oxan-2-yl]-3,4-dihydro-2H-1-benzopyran-2-yl}phenoxy)oxane-2-carboxylic acid | p330.07794 | 1.4333 | 4.58E-05 | higher |
| 19-Noraldosterone | p383.14346 | -2.7233 | 4.64E-05 | lower |
| N-lactoyl-Methionine | n220.06555 | -1.1306 | 4.66E-05 | lower |
| unidentified | p687.76697 | -1.3731 | 4.83E-05 | lower |
| 1-(5Z,8Z,11Z,14Z,17Z-eicosapentaenoyl)-2-nonadecanoyl-glycero-3-phospho-(1'-sn-glycerol) | p425.25446 | 1.6382 | 4.88E-05 | higher |
| dUDP | n426.95004 | -1.8907 | 5.24E-05 | lower |
| {2-[2-(3,4-dihydroxyphenyl)-5-hydroxy-7-methoxy-4-oxo-4H-chromen-6-yl]-3,5-dihydroxy-6-(hydroxymethyl)oxan-4-yl}oxidanesulfonic acid | p587.04285 | 2.1108 | 5.4E-05 | higher |
| Capsicoside E | n163.97862 | 2.6038 | 5.56E-05 | higher |
| [7,8,8,12,13,21-hexahydroxy-19-(hydroxymethyl)-3,6,16-trioxo-2,17,20,23-tetraoxapentacyclo[16.3.1.1â·,Â¹Â¹.0â´,â¹.0Â¹â°,Â¹âµ]tricosa-4,10,12,14-tetraen-22-yl]oxidanesulfonic acid | n598.99756 | 2.0894 | 5.59E-05 | higher |
| unidentified | p1156.55469 | 1.818 | 5.96E-05 | higher |
| (1E)-1-(4-hydroxy-3-methoxyphenyl)dec-1-en-3-one | n313.13684 | -1.8514 | 6.04E-05 | lower |
| unidentified | p634.99457 | 1.8574 | 6.14E-05 | higher |
| Androstanedione | p577.42633 | -2.2899 | 6.16E-05 | lower |
| Phenylalanyl-Valine | p133.08032 | -5.397 | 6.2E-05 | lower |
| unidentified | p282.84491 | 2.7329 | 6.26E-05 | higher |
| Dehydrocarpaine I | p250.17827 | 1.6571 | 6.38E-05 | higher |
| 1,3-bis(4-hydroxyphenyl)prop-2-en-1-one | p223.07498 | 1.921 | 6.65E-05 | higher |
| 1,3-dipentadecanoyl-2-(9Z-octadecenoyl)-glycerol (d5) | n848.71826 | 1.7687 | 6.73E-05 | higher |
| 2,3,4,7,8,9,15,22,23,28-decahydroxy-14-(hydroxymethyl)-13,25,32,35-tetraoxaoctacyclo[14.13.3.3Â¹â·,Â²â¶.0âµ,Â³â°.0â¶,Â¹Â¹.0Â¹â¸,Â²â¶.0Â¹â¹,Â²â´.0Â²â¹,Â³Â³]pentatriaconta-1,3,5(30),6,8,10,19(24),20,22-nonaene-12,27,31,34-tetrone | n356.04095 | -1.6258 | 6.79E-05 | lower |
| Glycyl-Valine | n173.09325 | 1.7239 | 6.84E-05 | higher |
| 4-(Methylnitrosamino)-1-(3-pyridyl)-1-butanol | p210.12422 | 1.8678 | 7.07E-05 | higher |
| 1,2-diheneicosanoyl-sn-glycero-3-phospho-(1'-myo-inositol) | n987.65509 | 2.1421 | 7.11E-05 | higher |
| TG(20:5(5Z,8Z,11Z,14Z,17Z)/20:5(5Z,8Z,11Z,14Z,17Z)/20:5(5Z,8Z,11Z,14Z,17Z)) | n979.66742 | 1.9464 | 7.32E-05 | higher |
| 3,4,5-trihydroxy-6-[3-(3,5,7-trihydroxy-4-oxo-4H-chromen-2-yl)phenoxy]oxane-2-carboxylic acid | n153.01944 | 1.7435 | 7.35E-05 | higher |
| (3beta,5alpha,6beta,22E,24R)-23-Methylergosta-7,22-diene-3,5,6-triol | n911.70172 | 2.0316 | 7.48E-05 | higher |
| Thyrotropin releasing hormone | p440.09427 | -1.426 | 7.57E-05 | lower |
| 1-tetracosyl-2-(8Z,11Z,14Z,17Z-eicosatetraenoyl)-sn-glycero-3-phosphocholine | n916.68323 | 2.4509 | 7.63E-05 | higher |
| unidentified | n733.85004 | 1.7949 | 7.77E-05 | higher |
| Thymidine 5'-triphosphate | p504.97894 | 1.3591 | 8.05E-05 | higher |
| 13'-Carboxy-gamma-tocopherol | p910.71118 | 2.0984 | 8.16E-05 | higher |
| unidentified | n277.92786 | -1.6911 | 8.33E-05 | lower |
| Guanosine triphosphate | p505.98788 | -1.4763 | 8.4E-05 | lower |
| unidentified | p60.62941 | 1.5499 | 8.63E-05 | higher |
| Methyl nomilinate 17-glucoside | p362.14624 | -1.7532 | 8.8E-05 | lower |
| Creatine riboside | p340.03122 | -1.565 | 9.13E-05 | lower |
| Glucosylceramide (d18:1/26:0) | p862.70471 | 2.5033 | 9.52E-05 | higher |
| KDNalpha2-3Galbeta1-4Glcbeta-Cer(d18:1/16:0) | p556.85266 | 1.7679 | 9.57E-05 | higher |
| 5-oxo-7-octenoic acid | p313.16412 | 0.88193 | 9.87E-05 | higher |
| 3,4,5-trihydroxy-6-{[4-oxo-1-(5,6,7-trimethoxy-4-oxo-4H-chromen-2-yl)cyclohexa-2,5-dien-1-yl]oxy}oxane-2-carboxylic acid | n541.09534 | -1.5184 | 9.93E-05 | lower |
| 3'-phosphoadenosine 5'-(3-{(3R)-3-hydroxy-2,2-dimethyl-4-[(3-{[2-(octacosanoylsulfanyl)ethyl]amino}-3-oxopropyl)amino]-4-oxobutyl} dihydrogen diphosphate) | p596.79376 | 1.836 | 0.000102 | higher |
| 3,4,5-trihydroxy-6-{[2-oxo-3-(3,4,5-trihydroxyphenyl)propanoyl]oxy}oxane-2-carboxylic acid | p465.98703 | -2.3814 | 0.000106 | lower |
| PE-NMe(24:0/24:1(15Z)) | p910.75934 | 1.7245 | 0.000106 | higher |
| 2-(Formamido)-N1-(5-phospho-D-ribosyl)acetamidine | n321.9855 | -2.4268 | 0.000107 | lower |
| 1,2-ditetradecanoyl-sn-glycero-3-phosphosulfocholine | p361.20734 | -1.8886 | 0.000117 | lower |
| [2-hydroxy-6-methoxy-4-(3,5,7-trihydroxy-5H-chromen-2-yl)phenyl]oxidanesulfonic acid | n431.99359 | -1.6312 | 0.000124 | lower |
| unidentified | p730.84412 | 1.8379 | 0.000126 | higher |
| 1-docosanoyl-glycero-3-phospho-(1'-myo-inositol) | p328.18417 | 1.4995 | 0.00013 | higher |
| unidentified | n720.77063 | 2.0261 | 0.000132 | higher |
| 1-O-hexadecanoyl-N-(hexadecanoyl)-sphing-4-enine | n812.7179 | 1.3242 | 0.000132 | higher |
| 1,3-dipentadecanoyl-2-(9Z-heptadecenoyl)-sn-glycerol (d5) | n834.70642 | 1.5417 | 0.000133 | higher |
| cholest-5,24-dien-3beta-ol(d6) | n780.75049 | 2.3107 | 0.000136 | higher |
| N-(2-hydroxyhexacosanoyl)-eicosasphinganine | n762.67902 | -1.5073 | 0.000136 | lower |
| 2-{3-[2,4-dihydroxy-3-(3-methylbut-2-en-1-yl)benzoyl]-4-(2,4-dihydroxyphenyl)-6-methyl-7-oxabicyclo[4.1.0]heptan-2-yl}-5-[(E)-2-(2,4-dihydroxyphenyl)ethenyl]benzene-1,3-diol | p356.11282 | 2.0349 | 0.00014 | higher |
| Estrone glucuronide | n446.19199 | -1.4475 | 0.000141 | lower |
| Tryptophyl-Tyrosine | p406.11508 | -1.7686 | 0.000145 | lower |
| {2-[2-(3,4-dihydroxyphenyl)-3,5,7-trihydroxy-4-[3,5,7-trihydroxy-2-(3-hydroxyphenyl)-3,4-dihydro-2H-1-benzopyran-8-yl]-3,4-dihydro-2H-1-benzopyran-6-yl]-3,5-dihydroxy-6-(hydroxymethyl)oxan-4-yl}oxidanesulfonic acid | p422.06308 | -1.7541 | 0.000147 | lower |
| 22-Dehydroclerosterol | n819.70795 | 2.0064 | 0.000151 | higher |
| TG(14:0/20:0/o-18:0) | p425.92795 | -4.4105 | 0.000152 | lower |
| 10-HETE | n359.19562 | -1.4319 | 0.000153 | lower |
| (10Z,14E,16E)-10,14,16-Octadecatrien-12-ynoic acid | p275.20078 | 1.5262 | 0.000155 | higher |
| {[5-(4-methoxyphenyl)-2-methyl-3-oxopent-4-en-1-yl]oxy}sulfonic acid | p318.0997 | 1.4139 | 0.000156 | higher |
| Galalpha1-3(GalNAcbeta1-4)Galbeta1-4Glcbeta-Cer(d18:1/18:0) | p647.88239 | -1.4872 | 0.000161 | lower |
| 2-(2,4-dihydroxyphenyl)-5-hydroxy-8-(hydroxymethyl)-8-methyl-4H,8H-pyrano[3,2-g]chromen-4-one | p369.0968 | 0.97298 | 0.000162 | higher |
| unidentified | n474.18689 | -1.148 | 0.000163 | lower |
| 1-pentadecanoyl-glycero-3-phosphate | p210.11227 | 1.2161 | 0.000167 | higher |
| unidentified | p774.82434 | 1.6371 | 0.00017 | higher |
| 12-hydroxy-5,8,10-heptadecatrienoic acid | n321.14542 | -1.5338 | 0.000172 | lower |
| 15,16-dihydroxy-11-methoxy-6,8,20-trioxapentacyclo[10.8.0.0Â²,â¹.0Â³,â·.0Â¹â´,Â¹â¹]icosa-1(12),2(9),10,14(19),15,17-hexaen-13-one | p365.06448 | -1.5611 | 0.000174 | lower |
| Neuraminic acid | n304.04431 | 0.030109 | 0.000176 | lower |
| 2-(3-hydroxyphytanyl)-3-phytanyl-sn-glycerol | p709.62708 | 1.0146 | 0.000179 | higher |
| unidentified | n667.86401 | -1.7435 | 0.00018 | lower |
| N-Ornithyl-L-taurine | p317.0152 | -0.59456 | 0.000186 | lower |
| Prolyl-Arginine | n308.13806 | -1.6439 | 0.000189 | lower |
| 2,3-Dihydro-4-(4-methoxyphenyl)-1H-phenalene-1,2,3-triol | p684.20563 | 1.2637 | 0.000196 | higher |
| hexatetracontanoic acid | p340.35916 | 1.519 | 0.000196 | higher |
| LysoPA(0:0/16:0) | n447.20764 | -1.1632 | 0.000196 | lower |
| 3,4,5-trihydroxy-6-({7-methoxy-4-oxo-2-phenyl-8-[3,4,5-trihydroxy-6-(hydroxymethyl)oxan-2-yl]-4H-chromen-5-yl}oxy)oxane-2-carboxylic acid | p304.08557 | 1.4472 | 0.000198 | higher |
| 2-{2-[(6-carboxy-3,4,5-trihydroxyoxan-2-yl)oxy]-6-hydroxy-4-[6-hydroxy-7-(3-methylbut-2-en-1-yl)-1-benzofuran-2-yl]phenyl}-4-(2,4-dihydroxyphenyl)-6-methyl-7-oxabicyclo[4.1.0]heptane-3-carboxylic acid | p395.09988 | -1.9457 | 0.000199 | lower |
| 3-Methoxy-4-hydroxyphenylglycol glucuronide | p362.11765 | -3.3501 | 0.000199 | lower |
| Galalpha1-3(Fucalpha1-2)Galbeta1-4Glcbeta-Cer(d18:1/20:0) | p632.883 | 1.1393 | 0.0002 | higher |
| {2-hydroxy-5-[(1E)-3-oxo-3-[(3,4,5,6-tetrahydroxyoxan-2-yl)methoxy]prop-1-en-1-yl]phenyl}oxidanesulfonic acid | n443.02618 | -1.8201 | 0.000204 | lower |
| unidentified | n353.34464 | 1.4489 | 0.000204 | higher |
| unidentified | p737.80731 | -1.2835 | 0.000219 | lower |
| unidentified | n525.92188 | -2.1178 | 0.000219 | lower |
| 2'-O-(alpha-D-Manp)-6'-O-(alpha-D-Manp-1->6-alpha-D-Manp)-(1,2-dioctadecanoyl-sn-glycero-3-phospho-1'-myo-inositol) | p675.87036 | -1.5365 | 0.000231 | lower |
| unidentified | p607.88153 | -1.4693 | 0.000233 | lower |
| Isoleucylproline | p495.2601 | 1.7401 | 0.000234 | higher |
| TG(18:4(6Z,9Z,12Z,15Z)/22:6(4Z,7Z,10Z,13Z,16Z,19Z)/22:6(4Z,7Z,10Z,13Z,16Z,19Z)) | n1006.6756 | 1.6897 | 0.000235 | higher |
| gamma-Glutamyl-S-methylcysteine sulfoxide | n320.0228 | 1.341 | 0.000256 | higher |
| Guanosine 3',5'-bis(diphosphate) | p642.92413 | 1.2416 | 0.000259 | higher |
| DG(24:1(15Z)/24:0/0:0) | n790.73566 | 1.673 | 0.000264 | higher |
| unidentified | p827.81696 | 1.1441 | 0.000265 | higher |
| PE(22:2(13Z,16Z)/24:1(15Z)) | p450.86658 | 2.0423 | 0.000265 | higher |
| unidentified | p318.8277 | 1.7549 | 0.000266 | higher |
| N-(26-hydroxyhexacosanoyl)-sphinganine | p714.71399 | 1.7422 | 0.000284 | higher |
| phosphatidylinositol-3,4,5-trisphosphate | p524.97852 | 1.7808 | 0.000285 | higher |
| 11-Oxo-androsterone glucuronide | p463.23215 | 1.1982 | 0.000305 | higher |
| Tetrahydro-2,5-furan-diacetic acid | p211.05797 | 1.3877 | 0.00031 | higher |
| 18alpha-Hydroxyglycyrrhetic acid | n995.6427 | 2.2042 | 0.000313 | higher |
| Selenocysteine | p360.91925 | 1.8948 | 0.000313 | higher |
| 1-(2,3,4-trihydroxy-5-methoxyphenyl)-2-(2,4,5-trihydroxyphenyl)propan-1-one | n167.03517 | 1.8311 | 0.000316 | higher |
| unidentified | n169.07439 | -1.9077 | 0.000316 | lower |
| unidentified | p743.81378 | 1.5317 | 0.000321 | higher |
| unidentified | p827.81427 | 1.8032 | 0.000322 | higher |
| 1-(2,4,6-trihydroxyphenyl)-3-(3,4,5-trimethoxyphenyl)prop-2-en-1-one | n347.0932 | 1.4799 | 0.000327 | higher |
| 12a-Hydroxy-3-oxocholadienic acid | p194.12935 | 1.4924 | 0.000337 | higher |
| Dihydroferulic acid 4-sulfate | n297.00558 | 1.8626 | 0.00035 | higher |
| 2,5-dihydroxy-5-(methylamino)-3,4-diphenylpentanoic acid | p360.11856 | 1.3209 | 0.000351 | higher |
| 1-(4-hydroxyphenyl)pentan-3-one | p179.10686 | 1.8711 | 0.000364 | higher |
| testosterone glucuronide | p465.2486 | 1.3154 | 0.00037 | higher |
| 2-amino-4-({1-[(carboxymethyl)-C-hydroxycarbonimidoyl]-2-{[3-hydroxy-2-methyl-4-({7-oxo-7H-furo[3,2-g]chromen-4-yl}oxy)butan-2-yl]sulfanyl}ethyl}-C-hydroxycarbonimidoyl)butanoic acid | p298.09381 | -1.1504 | 0.00037 | lower |
| CDP-DG(a-25:0/i-22:0) | n581.86462 | 1.8153 | 0.000373 | higher |
| 12-bromo-octadecanoic acid | n361.17474 | -1.32 | 0.000377 | lower |
| 11-[(2R,3S)-3-[2-amino-3-methyl-4-(2-methyl-1,3-thiazol-4-yl)but-3-en-1-yl]-2-methyloxiran-2-yl]-3,7-dihydroxy-4,4,6,8-tetramethyl-5-oxoundecanoic acid | p1068.62622 | 1.7419 | 0.000379 | higher |
| Tryptophyl-Serine | p309.15509 | 1.4023 | 0.000381 | higher |
| unidentified | n359.95432 | 1.8766 | 0.000382 | higher |
| unidentified | n669.73468 | 1.6062 | 0.000389 | higher |
| 6-bromo-eicosa-5E,9Z-dienoic acid | p425.14487 | 2.2752 | 0.000394 | higher |
| Benzeneacetamide-4-O-sulphate | n230.01266 | 1.1201 | 0.000394 | higher |
| 4-[3-(3,5-dihydroxyphenyl)oxiran-2-yl]benzene-1,2-diol | n299.01645 | 1.1749 | 0.000394 | higher |
| dUDP | p408.98141 | 2.1742 | 0.000398 | higher |
| 3-bromo-2E-heptenoic acid | p248.95992 | -1.9072 | 0.000403 | lower |
| Retinyl beta-glucuronide | p463.26837 | 1.7252 | 0.000407 | higher |
| 8-Hydroxy-5,6-octadienoic acid | n155.0714 | 1.5269 | 0.00041 | higher |
| N-(2-hydroxyeicosanoyl)-4R-hydroxysphinganine-1-O-[D-mannopyranosyl-alpha1-2-myo-inositol-1-phosphate] | n514.82214 | 1.6534 | 0.000413 | higher |
| 2-[2-hydroxy-4-methoxy-3-(sulfooxy)phenyl]propanoic acid | p312.05533 | -1.3128 | 0.000422 | lower |
| unidentified | n739.74878 | 1.461 | 0.000434 | higher |
| unidentified | n594.97711 | 1.1554 | 0.000446 | higher |
| 1-(11Z-eicosenoyl)-2-(8Z,11Z,14Z-eicosatrienoyl)-3-11Z-docosenoyl-sn-glycerol | p516.92493 | 2.3411 | 0.00045 | higher |
| 5,7-dihydroxy-2-(4-hydroxyphenyl)-8-(3,4,5-trihydroxyoxan-2-yl)-4H-chromen-4-one | p403.10178 | -1.8808 | 0.000454 | lower |
| 2-O-(4-O-Methyl-a-D-glucopyranuronosyl)-D-xylose | p385.07275 | -1.4764 | 0.000456 | lower |
| Oxalosuccinic acid/Oxalosuccinate | n211.99304 | -1.5624 | 0.000468 | lower |
| 2,8-dihydroxy-3-(3-oxo-1-phenylbutyl)-4H-chromen-4-one | p363.06223 | -1.0975 | 0.000478 | lower |
| unidentified | n428.94528 | -1.5996 | 0.000479 | lower |
| unidentified | n615.02924 | 0.84104 | 0.000481 | higher |
| Tryptophyl-Aspartate | p337.1499 | -1.6112 | 0.000482 | lower |
| 2-Methyl-4-oxopentanedioic acid | p207.01457 | 1.4368 | 0.000483 | higher |
| 1-Hydroxy-3,6,7-trimethoxy-2,8-diprenylxanthone | n438.20142 | -1.4802 | 0.000483 | lower |
| Urolithin B 3-O-glucuronide | p433.05005 | -1.2654 | 0.00049 | lower |
| 1-O-hexadecanoyl-N-(octadecanoyl)-sphing-4-enine | p400.88788 | -1.125 | 0.000496 | lower |
| Galalpha1-3Galbeta1-4Glcbeta-Cer(d18:1/26:1(17Z)) | p593.90472 | 1.754 | 0.0005 | higher |
| 3,4,5-trihydroxy-6-(2-{5-hydroxy-8,8-dimethyl-2-oxo-2H,8H-pyrano[2,3-f]chromen-4-yl}phenoxy)oxane-2-carboxylic acid | p467.13388 | 1.359 | 0.0005 | higher |
| Phosphatidylinositol-3,4,5-trisphosphate | p627.97125 | 1.6274 | 0.000502 | higher |
| 3-Methyl-3-phenylazetidine | p148.11229 | 1.8865 | 0.000503 | higher |
| unidentified | n683.7738 | 1.6994 | 0.000506 | higher |
| 1-eicosyl-2-methyl-sn-glycero-3-phosphocholine | p576.41467 | -2.0116 | 0.000518 | lower |
| 4-Hydroxyandrostenedione glucuronide | p433.22363 | 1.4147 | 0.000537 | higher |
| 6-{[(2Z)-3-(6,7-dimethoxy-2H-1,3-benzodioxol-5-yl)-2-hydroxyprop-2-enoyl]oxy}-3,4,5-trihydroxyoxane-2-carboxylic acid | p485.05258 | -1.4298 | 0.000538 | lower |
| Lysyl-Glutamine | p275.17166 | 1.1426 | 0.000545 | higher |
| N-(eicosanoyl)-1-beta-lactosyl-sphinganine | n919.68884 | 2.127 | 0.000551 | higher |
| 1-octadecanoyl-sn-glycero-3-phosphoserine | p272.17349 | -0.87182 | 0.000579 | lower |
| 1-(9Z,12Z-heptadecadienoyl)-2-(5Z,8Z,11Z,14Z,17Z-eicosapentaenoyl)-sn-glycerol | n661.44946 | -1.3833 | 0.000611 | lower |
| unidentified | p396.91278 | -1.2318 | 0.000614 | lower |
| 1-{3-[(3,3-dimethyloxiran-2-yl)methyl]-2,4,6-trihydroxyphenyl}-3-(4-hydroxyphenyl)propan-1-one | p436.06305 | -1.226 | 0.000614 | lower |
| Methyl 2-propenyl pentasulfide | n254.88512 | -1.1726 | 0.000615 | lower |
| 1-alkyl-2-acylglycerophosphoethanolamine | p319.97833 | -1.7921 | 0.000618 | lower |
| GalNAcbeta1-4(NeuGcalpha2-3)Galbeta1-4Glcbeta-Cer(d18:1/18:0) | p701.91132 | -1.413 | 0.000622 | lower |
| unidentified | n151.50282 | 2.0827 | 0.000639 | higher |
| 3,4,5-trihydroxy-6-{[3-(2,4,5-trihydroxyphenyl)prop-2-enoyl]oxy}oxane-2-carboxylic acid | p411.03275 | 1.4327 | 0.000647 | higher |
| unidentified | n777.7467 | 1.6389 | 0.000649 | higher |
| Galbeta1-3GalNAcbeta1-4(NeuAcalpha2-8NeuAcalpha2-8NeuAcalpha2-3)Galbeta1-4Glcbeta-Cer(d18:1/24:1(15Z)) | p1128.56409 | 1.5038 | 0.000651 | higher |
| [2-(3,4-dihydroxyphenyl)-5,6,7-trihydroxy-4-oxo-3,4-dihydro-2H-1-benzopyran-3-yl]oxidanesulfonic acid | p401.01746 | -1.2351 | 0.000655 | lower |
| 2-amino-4-({1-[(carboxymethyl)-C-hydroxycarbonimidoyl]-2-({3-hydroxy-6-[3-(4-hydroxyphenyl)-3-oxopropyl]-5-methoxy-2,2-dimethyl-3,4-dihydro-2H-1-benzopyran-4-yl}sulfanyl)ethyl}-C-hydroxycarbonimidoyl)butanoic acid | p340.13547 | -2.0655 | 0.000657 | lower |
| TG(14:0/18:3(6Z,9Z,12Z)/o-18:0) | n851.72156 | 2.2213 | 0.000658 | higher |
| 1-(9Z-pentadecenoyl)-glycero-3-phospho-(1'-myo-inositol) | p1130.5647 | 1.7556 | 0.00066 | higher |
| unidentified | p114.26133 | -1.7499 | 0.000667 | lower |
| unidentified | n574.80322 | 1.8695 | 0.000667 | higher |
| Inositol cyclic phosphate | n264.99725 | -0.86883 | 0.000675 | lower |
| 11beta,17,21-trihydroxypregn-4-ene-3,20-dione 21-hexanoate | p483.27039 | -0.32954 | 0.000675 | lower |
| unidentified | p665.90491 | 1.6689 | 0.000693 | higher |
| 2'-O-(alpha-D-Manp)-6'-O-(alpha-D-Manp-1->6-alpha-D-Manp-1->6-alpha-D-Manp-1->6-alpha-D-Manp)-(1-hexadecanoyl-2-octadecanoyl-sn-glycero-3-phospho-1'-myo-inositol) | p844.90131 | 1.6261 | 0.000699 | higher |
| unidentified | p565.97748 | -1.5314 | 0.000709 | lower |
| 1,8-Epoxy-p-menthan-4-ol glucoside | p333.18991 | -1.0497 | 0.000721 | lower |
| Uridine triphosphate | p522.93311 | 2.8387 | 0.000729 | higher |
| Methyl (9Z)-8'-oxo-6,8'-diapo-6-carotenoate | n390.17361 | -1.6621 | 0.000742 | lower |
| 3-Hydroxymethylantipyrine | p205.09776 | 1.1409 | 0.000759 | higher |
| GalNAcbeta1-4(NeuGcalpha2-3)Galbeta1-4Glcbeta-Cer(d18:1/20:0) | p725.92737 | 1.2888 | 0.000768 | higher |
| Uridine 5'-diphosphate | p442.96387 | -1.4393 | 0.000768 | lower |
| TG(8:0/8:0/8:0) | p959.75519 | -0.94672 | 0.000782 | lower |
| N-methylphenylalanine | p381.17752 | -1.2971 | 0.000788 | lower |
| unidentified | n561.88104 | -1.7948 | 0.000804 | lower |
| Threoninyl-Tyrosine | p321.08545 | -2.2093 | 0.000806 | lower |
| {[1-(7-hydroxy-2-oxo-2H-chromen-8-yl)-3-methylbut-3-en-2-yl]oxy}sulfonic acid | n361.01614 | 1.5551 | 0.000811 | higher |
| 2'-O-(alpha-D-Manp)-6'-O-(alpha-D-Manp-1->6-alpha-D-Manp)-(1-(9Z-nonadecenoyl)-2-(9Z-octadecenoyl)-sn-glycero-3-phospho-1'-myo-inositol) | p691.88251 | 1.6629 | 0.000812 | higher |
| 3,4,5-trihydroxy-6-{[3-hydroxy-2-(2-hydroxypropan-2-yl)-7-oxo-2H,3H,7H-furo[3,2-g]chromen-9-yl]oxy}oxane-2-carboxylic acid | p455.11929 | 2.7089 | 0.000815 | higher |
| 2-Amino-3,4-dihydroxypentanedioic acid | p397.05084 | 1.3537 | 0.000817 | higher |
| 11Z-Eicosenyl acetate | p700.63037 | 1.3943 | 0.00083 | higher |
| unidentified | p470.01801 | -1.2489 | 0.000832 | lower |
| Fucalpha1-2(Galalpha1-3)Galbeta1-4GlcNAcbeta1-3(Galalpha1-3Galbeta1-4GlcNAcbeta1-6)Galbeta1-4GlcNAcbeta1-3Galbeta1-4Glcbeta-Cer(d18:1/16:0) | n808.71741 | 1.7302 | 0.000842 | higher |
| 1-dodecanoyl-2-(5Z,8Z,11Z,14Z-eicosatetraenoyl)-sn-glycerol | p561.45215 | -1.5578 | 0.000854 | lower |
| 3-[3,4-dihydroxy-5-(2,3,4,5-tetrahydroxybenzoyloxy)benzoyloxy]-4,5-dihydroxybenzoic acid | n513.01813 | -1.1041 | 0.000862 | lower |
| 2'-O-(alpha-D-Manp)-6'-O-(alpha-D-Manp)-(1-hexadecanoyl-2-tetradecanoyl-sn-glycero-3-phospho-1'-myo-inositol) | p1090.60889 | 1.4299 | 0.000868 | higher |
| 5-Hydroxy-L-tryptophan | n439.16235 | 1.7749 | 0.000881 | higher |
| 6-bromo-5Z,9Z-heptacosadienoic acid | p988.62292 | 1.6771 | 0.000893 | higher |
| unidentified | p600.80493 | 1.7335 | 0.000893 | higher |
| 2-Ethyl-4-methylthiazole | p272.12448 | 1.918 | 0.000898 | higher |
| 3,4-Dimethyl-5-pentyl-2-furanoctanoic acid | p331.22589 | 1.342 | 0.000902 | higher |
| unidentified | p668.75854 | -1.5395 | 0.000904 | lower |
| unidentified | p774.82532 | -0.74751 | 0.000906 | lower |
| 3-O-(beta-D-glucopyranosyl)-stigmast-5-en-3beta-ol | p300.21884 | 1.752 | 0.000907 | higher |
| Hydroxyisonobilin | n400.12579 | 1.3327 | 0.000915 | higher |
| unidentified | p365.94229 | 1.6016 | 0.000928 | higher |
| [(1-oxo-3-phenylpropan-2-yl)oxy]sulfonic acid | n229.01768 | 1.3998 | 0.000929 | higher |
| {2-[5,7-dihydroxy-2-(3-methoxyphenyl)-4-oxo-4H-chromen-6-yl]-3-hydroxy-6-methyl-5-oxooxan-4-yl}oxidanesulfonic acid | p274.01788 | -5.6355 | 0.000946 | lower |
| Galabiosylceramide (d18:1/22:0) | p963.74854 | 1.4276 | 0.000946 | higher |
| Myo-inositol hexakisphosphate | n681.84088 | -1.5401 | 0.000975 | lower |
| DG(24:1(15Z)/24:0/0:0) | n791.74109 | 2.0001 | 0.000981 | higher |
| unidentified | p667.86792 | -1.1805 | 0.000989 | lower |
| p-Cresol sulfate | p232.98596 | -1.4034 | 0.000992 | lower |
| 3,4,5-trihydroxy-6-{2-[(1E)-3-(4-hydroxy-2-methoxyphenyl)prop-1-en-1-yl]phenoxy}oxane-2-carboxylic acid | n432.13965 | -1.2105 | 0.001013 | lower |
| 1-(11Z,14Z-eicosadienoyl)-2-eicosanoyl-glycero-3-phospho-(1'-sn-glycerol) | n414.7948 | -1.0717 | 0.001019 | lower |
| unidentified | n736.74463 | 1.7428 | 0.001021 | higher |
| unidentified | n654.80823 | 1.5007 | 0.001038 | higher |
| Glycyl-Cysteine | p179.04877 | 1.3047 | 0.001038 | higher |
| 3-[3-hydroxy-5-methoxy-4-(sulfooxy)phenyl]prop-2-enoic acid | p336.9772 | 1.4163 | 0.001044 | higher |
| (25S)-5alpha-cholestan-3beta,4beta,6alpha,7alpha,8beta,15beta,16beta,26-octol | n166.44746 | -1.1614 | 0.001055 | lower |
| 5a-Dihydrotestosterone sulfate | p411.14496 | 1.3046 | 0.001058 | higher |
| N-(2-hydroxyethyl-1,1,2,2-d4)-hexadecanamide | p342.27182 | 1.4458 | 0.001062 | higher |
| Amlaic acid | p693.05048 | -2.1439 | 0.001071 | lower |
| (S)-5'-Deoxy-5'-(methylsulfinyl)adenosine | n350.034 | 1.3821 | 0.001072 | higher |
| unidentified | n335.89337 | -1.3328 | 0.001075 | lower |
| 5-Amino-4-hydroxy-3-(phenylazo)-2,7-naphthalenedisulfonic acid | n445.99942 | -0.882 | 0.001088 | lower |
| Dihydro-3(2H)-thiophenone | p227.01804 | -0.6585 | 0.001093 | lower |
| 1,2-dihexadecanoyl-3-(9Z-heptadecenoyl)-sn-glycerol | n856.70471 | 1.5486 | 0.001093 | higher |
| 4,2'-Dihydroxy-4',6'-dimethoxychalcone 4-O-(5'''-O-p-cinnamoyl)-apiofuranosyl-(1'''->2'')-glucoside | p382.10226 | 1.0932 | 0.001112 | higher |
| 2-[3-(sulfooxy)phenyl]acetic acid | p310.92307 | 1.8291 | 0.001116 | higher |
| unidentified | n143.91704 | 1.8114 | 0.00112 | higher |
| unidentified | p623.85608 | -1.2299 | 0.001121 | lower |
| 2-[4-(1,2-dihydroxyethyl)-5,11,12,13-tetrahydroxy-8-oxo-3,7-dioxatricyclo[7.4.0.0Â²,â¶]trideca-1(13),9,11-trien-10-yl]-3,4,8,9,10-pentahydroxy-6-oxo-6H-benzo[c]chromene-1-carboxylic acid | p339.02124 | -1.0299 | 0.001122 | lower |
| CDP-DG(18:2(9Z,11Z)/a-25:0) | p572.81488 | 1.2658 | 0.001125 | higher |
| unidentified | p817.89337 | -0.6762 | 0.001131 | lower |
| N-Acetyl-9-O-lactoylneuraminic acid | p404.11575 | -1.0441 | 0.001135 | lower |
| unidentified | n818.70642 | 1.2522 | 0.001142 | higher |
| [2-hydroxy-5-(3-phenylpropanoyl)phenyl]oxidanesulfonic acid | p340.0835 | 1.04 | 0.001145 | higher |
| 3,4,5-trihydroxy-6-(2-{[2-methoxy-4-(prop-2-en-1-yl)phenoxy]carbonyl}phenoxy)oxane-2-carboxylic acid | n497.10425 | 1.5872 | 0.00115 | higher |
| p-Cresol glucuronide | n305.06357 | -1.141 | 0.001154 | lower |
| 3-Hydroxydodecanedioic acid | n281.11557 | -0.73978 | 0.001155 | lower |
| 2-cis,6-trans,10-trans-Geranylgeranyl diphosphate | p473.18259 | 1.1192 | 0.001157 | higher |
| Alliosterol 1-rhamnoside 16-galactoside | n741.43958 | 1.1281 | 0.001166 | higher |
| sn-caldito-1-phosphoethanolamine | n533.79126 | 1.4782 | 0.001168 | higher |
| TG(24:0/20:3n6/o-18:0) | p500.98456 | 1.8413 | 0.001169 | higher |
| exo-5,6-Dimethylbicyclo[2.2.1]hept-5-en-2-ol | p121.10149 | 0.95565 | 0.001189 | higher |
| 3-O-(6'-O-(11Z,14Z-eicosadienoyl)-beta-D-glucopyranosyl)-stigmast-5-en-3beta-ol | n867.69635 | 1.5214 | 0.001195 | higher |
| Thymine glycol | n79.01722 | 1.5998 | 0.001208 | higher |
| 1-(1Z-hexadecenyl)-2-(9Z-tetradecenoyl)-glycero-3-phospho-(1'-sn-glycerol) | n675.46387 | 1.1216 | 0.001214 | higher |
| 1-(9Z-pentadecenoyl)-2-(9Z-hexadecenoyl)-3-(9Z,12Z-heptadecadienoyl)-sn-glycerol | p400.34451 | 1.1281 | 0.001216 | higher |
| Dihydroferulic acid 4-sulfate | n137.0081 | 1.4996 | 0.001219 | higher |
| 1-(11Z-docosenoyl)-2-eicosanoyl-glycero-3-phospho-(1'-myo-inositol) | n473.82703 | 1.4867 | 0.001224 | higher |
| 5-Hydroxy-3,6,7-trimethoxy-3',4'-(methylenedioxy)flavone | n371.07663 | 1.479 | 0.001225 | higher |
| 4-Mercaptobutyl glucosinolate | n444.0025 | -0.95864 | 0.001241 | lower |
| 2'-O-(alpha-D-Manp)-6'-O-(alpha-D-Manp)-(1-heptadecanoyl-2-tetradecanoyl-sn-glycero-3-phospho-1'-myo-inositol) | p561.81525 | 0.93597 | 0.001256 | higher |
| TG(10:0/i-24:0/i-24:0) | p483.97397 | -1.3365 | 0.001275 | lower |
| PS(22:2(13Z,16Z)/24:1(15Z)) | p948.66687 | 1.3394 | 0.001296 | higher |
| 1-(beta-D-Glucopyranosyloxy)-3-octanone | n305.16187 | 2.0175 | 0.001296 | higher |
| 2-[3-(sulfooxy)phenyl]acetic acid | n486.98257 | -1.0113 | 0.001297 | lower |
| 3-Hexaprenyl-4,5-Dihydroxybenzoic acid | p563.41028 | -1.6268 | 0.001299 | lower |
| 1-(1Z-eicosenyl)-2-tetradecanoyl-glycero-3-phosphate | p350.24124 | 0.90017 | 0.001302 | higher |
| 10E,12E-tetradecadiene-4,6-diynoic acid | n431.22238 | 0.88127 | 0.001309 | higher |
| Isopentenyl pyrophosphate | n284.96082 | -1.1421 | 0.001316 | lower |
| 2-Methoxy-estra-1,3,5(10)-triene-3,17beta-diol 3-sulfate | p400.17902 | 1.3785 | 0.001327 | higher |
| Triethylene Glycol Monomethyl Ether | n329.19882 | 2.6764 | 0.001338 | higher |
| N-carbamoylglutamic Acid | p229.02249 | 1.4111 | 0.001359 | higher |
| unidentified | p688.89178 | -1.3886 | 0.001371 | lower |
| unidentified | n690.88959 | 1.1311 | 0.001377 | higher |
| 2,7-dimethyl-6-octenoic acid | p171.13802 | 1.0527 | 0.001384 | higher |
| 13-bromo-10R,11R-dichloro-7,11-dimethyl-3-methylene-4R-hydroxy-6E,8E,12E-tridecatrienoic acid | p433.99838 | -1.5359 | 0.001396 | lower |
| unidentified | p110.02171 | 1.3426 | 0.001417 | higher |
| 5'-Carboxy meloxicam | p336.01093 | 1.5888 | 0.001419 | higher |
| unidentified | n386.15106 | -1.3525 | 0.001424 | lower |
| unidentified | n750.73962 | 1.4478 | 0.001436 | higher |
| DG(14:1(9Z)/22:6(4Z,7Z,10Z,13Z,16Z,19Z)/0:0) | n647.43341 | 1.0541 | 0.001448 | higher |
| TG(14:0/15:0/o-18:0) | p801.7334 | 1.3064 | 0.001449 | higher |
| unidentified | p656.2052 | 1.2712 | 0.001453 | higher |
| (2R)-2-[(1R)-1-hydroxy-17-{(1R,2R)-2-[(2R,21R,22R)-21-hydroxy-22-methyltetracontan-2-yl]cyclopropyl}heptadecyl]hexacosanoic acid | n426.09991 | -0.07042 | 0.001454 | higher |
| 1-O-{hydroxy[(hydroxy{[(2E,6E,10E,14E,18E,22E,26E,30E,34E,38E)-3,7,11,15,19,23,27,31,35,39,43-undecamethyltetratetraconta-2,6,10,14,18,22,26,30,34,38,42-undecaen-1-yl]oxy}phosphoryl)oxy]phosphoryl}-alpha-D-galactopyranose | n543.83502 | 1.5995 | 0.001479 | higher |
| unidentified | p345.91711 | -1.042 | 0.001497 | lower |
| Kaempferol 3-glucuronide-7-sulfate | n580.98645 | 1.4057 | 0.001514 | higher |
| 2-amino-4-[(2-{[2-carboxy-2-hydroxy-1-(4-hydroxy-3-methoxyphenyl)ethyl]sulfanyl}-1-[(carboxymethyl)-C-hydroxycarbonimidoyl]ethyl)-C-hydroxycarbonimidoyl]butanoic acid | p282.05856 | 1.4024 | 0.001539 | higher |
| N-(hexacosanoyl)-1-beta-glucosyl-sphinganine | n878.6864 | 1.4791 | 0.001545 | higher |
| Thymidine 5'-triphosphate | p482.99475 | 1.3721 | 0.001549 | higher |
| 2-Propanoylthiazole | p181.98596 | 0.93151 | 0.001549 | higher |
| unidentified | p1182.49475 | 1.541 | 0.001572 | higher |
| 9,10,12,13-tetrabromo-octadecanoic acid | p619.90723 | -1.5481 | 0.001578 | lower |
| 2,3,7,9-tetrahydroxy-1-methyl-6H-benzo[c]chromen-6-one | n312.01746 | 1.6291 | 0.001607 | higher |
| Glycerylphosphorylethanolamine | p454.1062 | 2.6879 | 0.00161 | higher |
| Thiamine monophosphate | n380.04364 | 1.3893 | 0.001611 | higher |
| 6-{2-[3-(3,4-dihydroxyphenyl)-1-[2-(3,4-dihydroxyphenyl)-3,5,7-trihydroxy-3,4-dihydro-2H-1-benzopyran-8-yl]-2-hydroxypropyl]-5-hydroxyphenoxy}-3,4,5-trihydroxyoxane-2-carboxylic acid | n369.08975 | -1.063 | 0.00162 | lower |
| 1-(9Z-heptadecenoyl)-sn-glycero-3-phosphate | p223.1194 | 1.2746 | 0.00162 | higher |
| 1,2-di-(9Z,12Z-heptadecadienoyl)-3-(11Z,14Z-eicosadienoyl)-sn-glycerol | p880.74951 | 1.2326 | 0.001623 | higher |
| 6-({10-[(2R)-3-[2-amino-3-methyl-4-(2-methyl-1,3-thiazol-4-yl)but-3-en-1-yl]-2-methyloxiran-2-yl]-1-carboxy-6-hydroxy-3,3,5,7-tetramethyl-4-oxodecan-2-yl}oxy)-3,4,5-trihydroxyoxane-2-carboxylic acid | n700.31995 | -1.6759 | 0.001645 | lower |
| Imidazoleacetic acid ribotide | p170.03302 | -4.9337 | 0.001646 | lower |
| unidentified | n511.0213 | -0.76183 | 0.00165 | lower |
| 2,4-Dimethyladipic acid | n370.15619 | -0.50957 | 0.001671 | lower |
| 1-dodecanoyl-2-(5Z,8Z,11Z,14Z,17Z-eicosapentaenoyl)-3-(7Z,10Z,13Z,16Z,19Z-docosapentaenoyl)-sn-glycerol | p436.84512 | -1.7145 | 0.001681 | lower |
| 6-(2-amino-2-carboxyethyl)-4-hydroxybenzothiazole | p239.04883 | -1.1875 | 0.001685 | lower |
| 5'-Carboxy meloxicam | p420.97614 | -0.95437 | 0.001698 | lower |
| Methionine sulfoxide | n200.01524 | -0.87204 | 0.001717 | lower |
| Phosphoadenosine phosphosulfate | p547.94611 | -0.75238 | 0.001725 | lower |
| [(1-oxo-1H-isochromen-3-yl)methoxy]sulfonic acid | p297.96942 | 0.95046 | 0.001734 | higher |
| unidentified | n312.89703 | -1.7171 | 0.00174 | lower |
| Monofucosyllacto-N-hexaose | p632.71063 | 1.4559 | 0.001743 | higher |
| 3,5,3-triiodothyronine-4-sulfate | n128.01146 | -0.54387 | 0.001755 | lower |
| unidentified | n691.75867 | 1.2237 | 0.001757 | higher |
| unidentified | p427.88049 | 1.0709 | 0.001787 | higher |
| MG(21:0/0:0/0:0) | p818.74194 | 1.5084 | 0.001789 | higher |
| 1-Acetyl-4-isopropenylcyclopentene | p151.11211 | 1.1366 | 0.001791 | higher |
| UDP-4-dehydro-6-deoxy-D-glucose | p276.02835 | 0.95791 | 0.001792 | higher |
| SM(d18:0/24:0) | n838.68402 | 1.7142 | 0.00181 | higher |
| 1-(15Z-tetracosenoyl)-2-(1Z-octadecenyl)-sn-glycero-3-phosphocholine | n890.67816 | 1.3877 | 0.001822 | higher |
| [3-(3,5,7-trihydroxy-5H-chromen-2-yl)phenyl]oxidanesulfonic acid | p429.94501 | -0.96686 | 0.001827 | lower |
| Indoxyl sulfate | p450.01297 | -1.3173 | 0.001829 | lower |
| 1-(9Z,12Z-heptadecadienoyl)-glycero-3-phosphoethanolamine | p944.57526 | -0.49031 | 0.001833 | lower |
| 1-tetradecanoyl-sn-glycero-3-phosphate | n403.18597 | -1.4088 | 0.001852 | lower |
| O-b-D-Gal-(1->3)-O-[O-b-D-Gal-(1->4)-2-(acetylamino)-2-deoxy-b-D-Glc-(1->6)]-2-(acetylamino)-2-deoxy- D-Galactose | p375.14581 | 1.2142 | 0.001856 | higher |
| unidentified | p686.98633 | 1.3744 | 0.001859 | higher |
| unidentified | n392.90036 | -0.44482 | 0.001863 | lower |
| Muramic acid | p328.02374 | -1.0532 | 0.001865 | lower |
| N-(9Z-octadecenoyl)-leucine | p350.34378 | 1.2003 | 0.00188 | higher |
| 3,4,5-trihydroxy-6-{[4-oxo-1-(5,6,7-trimethoxy-4-oxo-4H-chromen-2-yl)cyclohexa-2,5-dien-1-yl]oxy}oxane-2-carboxylic acid | p597.03827 | 1.0306 | 0.001882 | higher |
| 1-hydroxy-1-(4-methoxyphenyl)-4-methylpentan-3-one | p447.25381 | -0.14327 | 0.001894 | lower |
| 1-O-beta-D-Glucopyranosyl-2,3-di-O-(8-hexadecenoyl)glycerol | n725.51752 | -0.87879 | 0.001918 | lower |
| unidentified | n717.76764 | 1.0034 | 0.00194 | higher |
| unidentified | n318.00916 | -1.6651 | 0.001942 | lower |
| CDP-DG(a-13:0/a-15:0) | n298.81314 | -1.2375 | 0.001943 | lower |
| 1-(2,4-dihydroxy-5-methoxyphenyl)-2-(2,3,5-trihydroxy-4-methoxyphenyl)propan-1-one | p373.09076 | -0.62844 | 0.001955 | lower |
| {2-[5,7-dihydroxy-2-(4-hydroxyphenyl)-4-oxo-4H-chromen-6-yl]-6-methyl-5-oxo-3-[(3,4,5-trihydroxy-6-methyloxan-2-yl)oxy]oxan-4-yl}oxidanesulfonic acid | n319.04755 | 2.438 | 0.001961 | higher |
| Campesterol 6'-hexadecanoylglucoside | p801.65973 | 1.2174 | 0.001981 | higher |
| TG(16:0/22:5(4Z,7Z,10Z,13Z,16Z)/o-18:0) | p448.90921 | 1.1582 | 0.002013 | higher |
| unidentified | n451.92578 | -1.2594 | 0.002014 | lower |
| 5beta-Cholestane-3beta,7alpha,25-triol | p497.27731 | 1.7964 | 0.002024 | higher |
| 16-a-Hydroxypregnenolone | p372.20126 | 1.1811 | 0.002025 | higher |
| unidentified | n334.58948 | -1.5386 | 0.002032 | lower |
| unidentified | n57.97565 | 1.2177 | 0.002052 | higher |
| unidentified | p328.14954 | -0.65779 | 0.002053 | lower |
| 1-(9Z,12Z-heptadecadienoyl)-2-(6Z,9Z,12Z,15Z-octadecatetraenoyl)-3-(5Z,8Z,11Z,14Z-eicosatetraenoyl)-sn-glycerol | p443.85205 | 0.91855 | 0.00206 | higher |
| unidentified | p624.72412 | 1.1773 | 0.002088 | higher |
| (2R,6x)-7-Methyl-3-methylene-1,2,6,7-octanetetrol 2-glucoside | n182.08812 | -0.8517 | 0.002092 | lower |
| Lysyl-Methionine | p324.11191 | -2.8427 | 0.002095 | lower |
| 1-(9Z-tetradecenoyl)-glycero-3-phosphate | p761.39655 | -0.96918 | 0.002098 | lower |
| xanthurenic acid 8-O-sulfate | p327.9599 | 1.1111 | 0.002102 | higher |
| 2-{4,6-dihydroxy-2-methoxy-3-[3-methyl-4-(sulfooxy)but-2-en-1-yl]phenyl}acetic acid | n363.0563 | -0.54771 | 0.002113 | lower |
| unidentified | p692.77448 | 1.3261 | 0.002116 | higher |
| 12-hydroxypodocarpa-8,11,13-trien-16-oic acid | n295.13141 | 1.2269 | 0.00212 | higher |
| Glucosylceramide (d18:1/24:1(15Z)) | p793.67157 | 1.118 | 0.002131 | higher |
| 3-methyl-4-oxo-1,2-diphenylbutan-2-yl propanoate | p349.12067 | 1.4779 | 0.002133 | higher |
| Diguanosine diphosphate | p374.03702 | -0.76972 | 0.002141 | lower |
| 1-(11Z-docosenoyl)-2-(7Z,10Z,13Z,16Z-docosatetraenoyl)-glycero-3-phosphate | p824.61517 | -0.99832 | 0.002146 | lower |
| 2-trans-9,12,15,18-all-cis-Tetracosapentaenoyl-CoA | n368.12332 | 1.3946 | 0.002149 | higher |
| unidentified | p275.95456 | -1.093 | 0.00215 | lower |
| 1-Methoxy-2-methylbenzene | p140.10725 | 1.714 | 0.002155 | higher |
| Oxalosuccinic acid/Oxalosuccinate | p212.99994 | -1.0386 | 0.002162 | lower |
| PE(22:0/24:1(15Z)) | p932.69305 | 1.7916 | 0.002164 | higher |
| unidentified | n646.75531 | -0.72825 | 0.002177 | lower |
| unidentified | p567.82269 | 1.046 | 0.002179 | higher |
| {[4-(4-hydroxy-3-methoxyphenyl)butan-2-yl]oxy}sulfonic acid | p299.05676 | -0.95731 | 0.002179 | lower |
| N-(hexacosanoyl)-1-beta-glucosyl-sphinganine | n862.71265 | 1.6968 | 0.002189 | higher |
| 6,8-dihydroxy-octanoic acid | p372.24039 | 1.2344 | 0.002202 | higher |
| 2,5-Dichloro-4-oxohex-2-enedioate | n474.86707 | -0.65365 | 0.002204 | lower |
| (2E)-3-(2,3-dihydroxyphenyl)prop-2-enoic acid | n218.99709 | 1.1459 | 0.002228 | higher |
| Threoninyl-Glutamine | p202.1189 | 1.3441 | 0.002249 | higher |
| unidentified | n396.94589 | 1.9142 | 0.002263 | higher |
| Methyl 4-chloro-1H-indole-3-acetate | p301.9541 | -1.4419 | 0.002265 | lower |
| Melanin | p364.03979 | 1.083 | 0.002266 | higher |
| 4-{[4-({4-[(4-aminobutyl)(hydroxy)amino]-4-oxobutanoyl}amino)butyl](hydroxy)amino}-4-oxobutanoic acid | p429.17288 | 0.8089 | 0.002286 | lower |
| D-Linalool 3-(6''-malonylglucoside) | p385.1853 | 0.92381 | 0.002288 | higher |
| unidentified | p603.93274 | -0.97985 | 0.002292 | lower |
| unidentified | n396.79068 | -1.3813 | 0.002304 | lower |
| 6-Methoxy-9H-carbazole-3-carboxaldehyde | n450.15414 | -1.2415 | 0.00231 | lower |
| 1-Benzyloxy-1-(2-methoxyethoxy)ethane | p421.26001 | 0.80548 | 0.002337 | higher |
| 2'-O-(alpha-D-Manp)-6'-O-(alpha-D-Manp-1->6-alpha-D-Manp-1->6-alpha-D-Manp)-(1-octadecanoyl-sn-glycero-3-phospho-1'-myo-inositol) | n414.84586 | -1.0861 | 0.002343 | lower |
| unidentified | n403.95004 | -0.79865 | 0.002346 | lower |
| 4-[(2-Hydroxy-1-naphthalenyl)azo]benzenesulfonic acid | n363.0228 | 1.2731 | 0.002362 | higher |
| unidentified | n480.76001 | 1.2476 | 0.002366 | higher |
| 2-O-Galloyl-1,4-galactarolactone | n343.02936 | -0.92515 | 0.002375 | lower |
| 1-O-eicosanoyl-N-(octadecanoyl)-sphing-4-enine | p861.85986 | 1.0455 | 0.002404 | higher |
| 2'-O-(alpha-D-Manp)-6'-O-(alpha-D-Manp-1->6-alpha-D-Manp)-(1-(9Z,12Z-octadecadienoyl)-2-tetradecanoyl-sn-glycero-3-phospho-1'-myo-inositol) | p647.83459 | 0.90994 | 0.002408 | higher |
| unidentified | p666.91016 | -1.3515 | 0.00246 | lower |
| unidentified | p558.89386 | 1.1195 | 0.002468 | higher |
| 6-(2-{3-[2,4-dihydroxy-3-(3-methylbut-2-en-1-yl)benzoyl]-4-(2,4-dihydroxyphenyl)-6-methyl-7-oxabicyclo[4.1.0]heptan-2-yl}-5-[(E)-2-(2,4-dihydroxyphenyl)ethenyl]-3-hydroxyphenoxy)-3,4,5-trihydroxyoxane-2-carboxylic acid | p422.14532 | -0.96144 | 0.002469 | lower |
| 2-[2,4,6-trihydroxy-3-(3-methylbut-2-en-1-yl)phenyl]acetic acid | n291.05585 | 1.1069 | 0.002472 | higher |
| 1-(9Z-hexadecenoyl)-glycero-3-phosphoserine | n516.23376 | -1.1681 | 0.002475 | lower |
| unidentified | p372.90692 | -0.84673 | 0.002484 | lower |
| 2-Methoxyacetaminophen glucuronide | p402.07889 | -1.8537 | 0.002488 | lower |
| ({1-[2-(2,4-dihydroxyphenyl)-5,7-dihydroxy-6-(3-methylbut-2-en-1-yl)-4-oxo-3,4-dihydro-2H-1-benzopyran-3-yl]-3,7-dimethylocta-2,6-dien-4-yl}oxy)sulfonic acid | p296.10559 | 0.88902 | 0.002491 | higher |
| CDP-DG(18:2(9Z,11Z)/i-22:0) | p1106.58289 | 1.1136 | 0.002492 | higher |
| Tyrosyl-Tryptophan | n408.10684 | -0.96304 | 0.002516 | lower |
| 9-pentadecen-1-ol | p304.15213 | 1.2817 | 0.002533 | higher |
| 9S,11R-dihydroxy-15-oxo-2,3,4,5-tetranor-prostan-1,20-dioic acid | p331.1748 | 1.2352 | 0.002535 | higher |
| [2-(methoxymethyl)-5-(3,5,7-trihydroxy-3,4-dihydro-2H-1-benzopyran-2-yl)phenyl]oxidanesulfonic acid | p200.04106 | 2.0256 | 0.00255 | higher |
| 5-Hydroxyindoleacetylglycine | n82.02972 | 1.0851 | 0.002553 | higher |
| unidentified | p699.85065 | -0.83283 | 0.002558 | lower |
| 3-Mercaptopropanoic acid | p61.01105 | 1.9409 | 0.002596 | higher |
| (2E)-4-hydroxy-3-methylbut-2-en-1-yl trihydrogen diphosphate | p525.00684 | 0.88226 | 0.002604 | higher |
| Valerylcarnitine | p269.15619 | 1.1809 | 0.002621 | higher |
| unidentified | p764.75983 | -1.1592 | 0.002623 | lower |
| PE(24:0/P-18:1(11Z)) | p798.65411 | 1.0595 | 0.002624 | higher |
| unidentified | p735.80908 | -0.84594 | 0.002626 | lower |
| unidentified | p334.89542 | -1.427 | 0.002637 | lower |
| 1-(9Z-hexadecenoyl)-glycero-3-phosphate | p453.19781 | -0.46392 | 0.002701 | lower |
| Phosphatidylserine | p426.07535 | 0.70224 | 0.002721 | higher |
| unidentified | p632.77551 | 1.2553 | 0.002737 | higher |
| Galabiosylceramide (d18:1/9Z-18:1) | p890.64447 | 1.1359 | 0.002741 | higher |
| 2-Aminobenzoic acid | p178.00897 | 1.1176 | 0.002784 | higher |
| 1-(2,4-dihydroxyphenyl)-3-{3-[(3,3-dimethyloxiran-2-yl)methyl]-4-hydroxyphenyl}prop-2-en-1-one | n339.12341 | -5.303 | 0.002788 | lower |
| unidentified | p654.86438 | 1.1181 | 0.002794 | higher |
| 2-Aminonicotinic acid | p316.05328 | 1.2642 | 0.002811 | higher |
| 1-(11Z-docosenoyl)-2-heneicosanoyl-glycero-3-phospho-(1'-myo-inositol) | p482.85004 | 1.1705 | 0.002821 | higher |
| 2-{[hydroxy(4-hydroxy-1H-indol-3-yl)methylidene]amino}acetic acid | n467.12085 | 1.5465 | 0.002856 | higher |
| 10,13-dimethyltetradecanoic acid | p295.20309 | 0.98577 | 0.002863 | higher |
| unidentified | p320.92038 | -1.0744 | 0.002888 | lower |
| Ganglioside GD3 (d18:1/12:0) | p713.86432 | 1.613 | 0.002889 | higher |
| methyl 9-oxo-11R-hydroxy-15R-acetoxy-5Z,13E-prostadienoate | p409.25653 | 1.1748 | 0.002893 | higher |
| Fucalpha1-2Galalpha1-3Galalpha1-4Galbeta1-4Glcbeta-Cer(d18:1/26:1(17Z)) | p757.95288 | 0.83917 | 0.002903 | higher |
| Dehydroascorbic acid | p389.00134 | -1.4365 | 0.002906 | lower |
| 2-Aminobenzoic acid | p139.05846 | -1.3879 | 0.002923 | lower |
| 7-Aminomethyl-7-carbaguanine | p359.16916 | 0.83344 | 0.002999 | higher |
| unidentified | p344.94482 | 1.6164 | 0.003027 | higher |
| 3'-N-Acetyl-4'-O-(9-octadecenoyl)fusarochromanone | p322.18909 | -0.74705 | 0.003032 | lower |
| Cysteineglutathione disulfide | p465.05307 | -0.5149 | 0.003036 | lower |
| 2-S-cysteinyl-DOPA | p392.99179 | -0.65018 | 0.003052 | lower |
| unidentified | p322.83701 | 2.1347 | 0.003099 | higher |
| unidentified | p652.78461 | -1.083 | 0.003104 | lower |
| 3-{[(9Z)-17-carboxyheptadec-9-enoyl]oxy}-4-(trimethylazaniumyl)butanoate | p928.68439 | -0.78117 | 0.003234 | lower |
| 3,4,5-trihydroxy-6-({7-oxo-7H-furo[3,2-g]chromen-4-yl}oxy)oxane-2-carboxylic acid | n416.02701 | -0.56101 | 0.003248 | lower |
| Methyl methanethiosulfonate | p276.95441 | -1.0477 | 0.0033 | lower |
| 2-{[hydroxy(4-hydroxy-3,5-dimethoxyphenyl)methylidene]amino}acetic acid | p298.03253 | 1.2351 | 0.003312 | higher |
| 8,8-Dimethyl-2-phenyl-4H,8H-benzo[1,2-b:3,4-b']dipyran-4-one | p172.04024 | 0.87805 | 0.003353 | higher |
| {2-[2-(3,4-dihydroxyphenyl)-5,7-dihydroxy-4-oxo-4H-chromen-6-yl]-3,5-dihydroxy-6-(hydroxymethyl)oxan-4-yl}oxidanesulfonic acid | p530.06702 | -0.84087 | 0.003365 | lower |
| DG(16:0/0:0/22:5n3) | p681.48584 | 0.98534 | 0.003391 | higher |
| (5-ethyl-2-hydroxyphenyl)oxidanesulfonic acid | p294.94437 | 0.853 | 0.003419 | higher |
| 3,4,5,13,21,22,23-heptahydroxy-8,18-dioxo-12-(3,4,5-trihydroxybenzoyloxy)-9,14,17-trioxatetracyclo[17.4.0.0Â²,â·.0Â¹â°,Â¹âµ]tricosa-1(23),2,4,6,19,21-hexaen-11-yl 3,4,5-trihydroxybenzoate | p394.05209 | -1.8572 | 0.00343 | lower |
| 16-hydroxy-18-bromo-8E,17E19Z-tricosatrien-4,6-diynoic acid | p453.18481 | 0.23352 | 0.003444 | lower |
| 13-(beta-D-glucopyranosyloxy)docosanoic acid | p501.3793 | 0.94073 | 0.003451 | higher |
| 2-amino-4-({1-[(carboxymethyl)-C-hydroxycarbonimidoyl]-2-{[1-(3,4-dimethoxyphenyl)-2-{[2-(3,4-dimethoxyphenyl)ethyl]-C-hydroxycarbonimidoyl}-2-hydroxyethyl]sulfanyl}ethyl}-C-hydroxycarbonimidoyl)butanoic acid | p367.11136 | -1.648 | 0.003479 | lower |
| unidentified | p860.87708 | 1.1018 | 0.00348 | higher |
| (+/-)N-(2-fluro-ethyl)-2,16,16-trimethyl-5Z,8Z,11Z,14Z-docosatetraenoyl amine | p840.72437 | 1.2128 | 0.003484 | higher |
| Fucalpha1-2Galalpha1-3Galalpha1-4Galbeta1-4Glcbeta-Cer(d18:1/16:0) | p685.87451 | 1.2666 | 0.003527 | higher |
| unidentified | p614.84045 | -1.0796 | 0.00353 | lower |
| NeuAcalpha2-3Galbeta-Cer(d18:1/26:0) | p588.90143 | 1.2106 | 0.003556 | higher |
| [3-(6,7-dimethoxy-2H-1,3-benzodioxol-5-yl)propoxy]sulfonic acid | p397.98627 | -0.44584 | 0.003973 | lower |
| 2-bromobutanedioic acid;2-bromosuccinic acid | p394.88968 | -1.7099 | 0.004528 | lower |
